# Sub-mV tunable photonic p-bits for probabilistic computing

**DOI:** 10.1126/sciadv.aeb9277

**Published:** 2026-05-15

**Authors:** Juhyung Seo, Taehyun Park, Jun-Young Park, Han-Koo Lee, Jae Yeon Park, Wonjun Shin, Joon-Kyu Han, Hocheon Yoo

**Affiliations:** ^1^ Department of Electronic Engineering, Hanyang University, Seoul 04763, Republic of Korea.; ^2^ Department of Materials Science and Engineering and Inter-University Semiconductor Research Center (ISRC), Seoul National University, Seoul 08826, Republic of Korea.; ^3^ Pohang Accelerator Laboratory, Pohang University of Science and Technology (POSTECH), Pohang 37673, Republic of Korea.; ^4^ Radiation Fusion Technology Research Division, Advanced Radiation Technology Institute (ARTI)/Korea Atomic Energy Research Institute (KAERI), Jeongeup 56212, Republic of Korea.; ^5^ Department of Semiconductor Convergence Engineering, Sungkyunkwan University, Suwon 16419, Republic of Korea.; ^6^ Department of Artificial Intelligence Semiconductor Engineering, Hanyang University, Seoul 04763, Republic of Korea.

## Abstract

Randomness, once dismissed as unwanted noise, is now emerging as a foundation for intelligent computation. Probabilistic bits (p-bits), which fluctuate between 0 and 1 with tunable probability, offer a route to solve complex problems through stochastic logic and energy-based optimization. Here, we present light-induced bias-tunable probabilistic-bit (LBP-bit) devices that generate entropy through light-induced charge polarity switching in a back-to-back junction. The probability of each device’s stochastic bitstream can be precisely tuned with submillivolt bias without disturbing the underlying distribution. This unique separation of randomness generation (by light) and probability control (by bias) enables stable control of output probability, essential for scalable probabilistic computing (p-computing). The proposed p-computing framework demonstrates integer factorization as a representative example of probabilistic search on computationally intensive problems. Max-Cut problems, representative combinatorial optimization tasks, are evaluated, demonstrating that light in this device functions as the stochastic source enabling probabilistic computation.

## Teaser

A light-induced and bias-controlled p-bit enables a probabilistic computing system with tunable randomness.

## INTRODUCTION

As modern computing faces growing demands from optimization, inference, and machine learning, the limits of deterministic logic and von Neumann architectures are becoming clear (*1*–*4*). Quantum computing has emerged as a promising alternative (*5*–*7*), offering exponential speedup for specific problems through superposition and entanglement. Yet, its practical use remains constrained by limited scalability and environmental requirements such as cryogenic condition (*8*, *9*).

This has sparked growing interest in alternative computing frameworks that provide similar advantages. Probabilistic computing (p-computing) is one such approach, relying on tunable randomness to explore vast solution spaces and avoid getting trapped in local minima (*10*–*14*). These properties are particularly valuable for solving combinatorial and inference-driven problems (*10*, *15*). At the heart of this approach lies the probabilistic bit (p-bit), a classical counterpart to the quantum bit (q-bit). While a q-bit exists in a fragile superposition of states until measured, a p-bit fluctuates continuously between 0 and 1 in real time, with its probability of switching controlled by an external input (*16*, *17*). If a q-bit is like a coin spinning in a sealed box, undecided until observed, then a p-bit is like a biased coin that keeps flipping in front of you, where you can adjust the weight to favor heads or tails (*18*–*20*). This makes p-bits more robust, transparent, and practical for hardware implementation. They enable invertible logic computation, energy-based optimization, and solving nondeterministic problems.

Recent photonic approaches have also demonstrated that light can serve as both the source of stochasticity and the means of probability control (*12*, *21*–*24*). These efforts include photonic p-bits driven by quantum-vacuum fluctuations in degenerate optical parametric oscillators (OPOs), probabilistic processors based on chaotic light, a photonic p-bit realized through vacuum-level optical biasing of a single OPO, and stochastic logic implemented in coupled and biased photonic p-bit networks. These platforms offer attractive features such as high integration density and energy efficiency, while many existing p-bit devices rely on a single physical quantity (typically, voltage, current, or lights).

To realize this concept of the p-bit, a variety of physical implementations have been proposed, including stochastic magnetic junctions (*18*, *19*, *25*), diffusive memristors (*26*–*29*), and bistable resistors (*30*). These platforms offer attractive features such as high integration density and energy efficiency. While many existing p-bit devices rely on a single physical quantity (typically, voltage or current) to both generate and control stochastic behavior, efforts to physically decouple these two functions have been relatively underexplored. In such conventional approaches, the same signal pathway is responsible for initiating randomness and tuning its output bias, potentially introducing constraints on precision or tunability (*31*, *32*). In contrast, decoupling the physical mechanisms for stochastic generation and probabilistic control offers several architectural advantages: It allows for more precise tuning of output distributions without disrupting the underlying randomness, reduces sensitivity to environmental fluctuations, and enhances scalability by isolating control signals from noise sources (*31*, *33*). Despite these potential benefits, such decoupled designs have rarely been attempted.

**Fig. 1.**
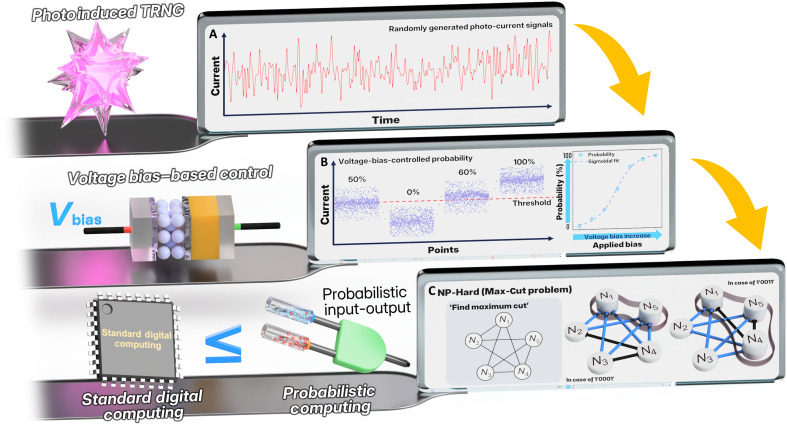
Conceptual diagram of p-bit generation, control, and application. (**A**) Generation of p-bits from analog signals induced by optical input. (**B**) Bias-driven modulation for quantitative control of p-bit behavior. (**C**) Demonstration of Max-Cut problem solving as an application of controlled p-bit outputs.

Motivated by the unexplored potential of decoupled p-bit architectures, we propose a unpreceded approach of p-bit device in which the generation and control of output probability are modulated by independent physical inputs, as shown in Fig. 1. The proposed approach is based on a back-to-back heterojunction structure that exhibits both wavelength-selective photoresponse and voltage-tunable current asymmetry. In this structure, two serially connected junctions with distinct optical response characteristics are selectively activated depending on the wavelength of the incident light. Upon illumination, photogenerated electron-hole pairs are formed within the activated junction and separated by the built-in potential, resulting in a photocurrent with either positive or negative polarity. When the device is driven by a stochastic polychromatic light source, the dominant junction changes randomly over time, and the direction of the photocurrent fluctuates accordingly producing a random bitstream composed of 0 and 1 s. The built-in potentials of the oppositely arranged heterojunctions respond in opposite directions under a single applied bias, effectively realizing structural independence between the random generation and control pathways without altering the fundamental stochastic generation process. Above-described shift in the photocurrent distribution takes form of a sigmoidal probability response, in which the average switching probability is smoothly tunable by bias while preserving the shape of the underlying distribution. This decoupling of randomness generation and probability control enables more robust and flexible operation in p-computing contexts.

Based on the measured sigmoid response of our device, we perform its applicability in representative p-computing tasks. Using experimentally derived probability data as input, we simulate an invertible multiplier, AND logic, and Max-Cut solving, demonstrating the practical potential of the proposed architecture for probabilistic inference and optimization problems. In these tasks, the proposed system achieves a solution accuracy of 0.80 in integer factorization. For the Max-Cut problem, it attains an average solution accuracy of 0.72 across four different five-node instances, values that exceed the pass threshold of 0.2 by more than a factor of 3 and highlight the ideal operation of p-computing.

## RESULTS

**Fig. 2.**
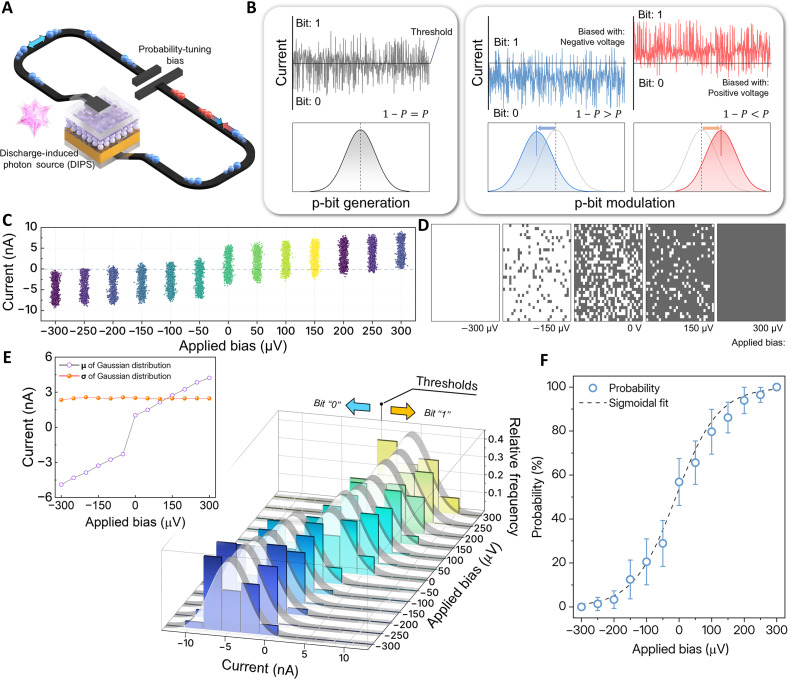
Schematic and probabilistic characteristics of the LBP-bit generator. (**A**) Schematic diagram of the LBP-bit generator, comprising a DIPS light source, the multiheterojunction device, and a probability-tuning electrical bias input. (**B**) Bias-dependent analog signals and corresponding Gaussian distributions of the LBP-bit generator under illumination. (**C**) Output current points measured under illumination in response to sequential voltage-bias steps from −300 to +300 μV in 50-μV increments. (**D**) Modulation of p-bit mappings across a 32 by 32 array in response to control-bias variation, with output probabilities tuned from 0 to 100%. (**E**) Gaussian distributions of current signal data points generated by the LBP-bit generator, and the bias-dependent shift of the Gaussian mean. (**F**) Sigmoidal curve representing the probability modulated by the applied control voltage bias.

Figure 2 illustrates the configuration of the designed p-bit generating system and the characteristics of its controlled output. The system is composed of two primary parts: a light-induced bias-tunable probabilistic-bit (LBP-bit) generator, which transforms the received optical stimulus into a stream of random analog signal, and a discharge-induced photon source (DIPS) that provides the optical input. The LBP-bit generator is further subdivided into the main device containing multiheterojunction layers and a voltage bias module for probability tuning. As shown in Fig. 2A, the proposed device is fabricated in a stacked structure, which consists of a bottom indium tin oxide (ITO) electrode, a poly(9,9-dioctylfluorene-alt-benzothiadiazole) (F8BT) (*34*, *35*) organic semiconductor layer, a tin dioxide quantum dots (SnO_2_ QDs) (*36*, *37*) layer, and a top poly(3,4 ethylenedioxythiophene) polystyrene sulfonate (PEDOT:PSS) (*38*, *39*) electrode. Detailed material characterization, including x-ray photoelectron spectroscopy (XPS) and x-ray absorption spectroscopy (XAS), is presented in supplementary text 1 and fig. S1. The stacking of these layers forms a pn junction between F8BT and the SnO_2_ QDs, as well as a metal-semiconductor (ms) junction between the SnO_2_ QDs and the PEDOT:PSS electrode. This multiheterojunction architecture is fundamental to the generation of random currents via multiple photoabsorption processes at the device level. This behavior is analyzed in detail in the subsequent section concerning the device mechanism.

As illustrated in Fig. 2B, the LBP-bit generator exhibits distinct behaviors under optical illumination depending on the applied voltage bias. Under zero bias, the device basically generates a photocurrent through the photovoltaic effect in its illuminated junctions. This photocurrent yields ideal p-bits with a symmetric Gaussian distribution, in which the probability of producing a “1” (denoted as *P*) is 0.5 (i.e., 1 − *P* = *P*). When a negative bias is applied, the mean of the distribution shifts to the left. This shift results in a larger fraction of the output currents falling below the fixed threshold, thereby increasing the generation probability of a “0” bit, such that *P* < 0.5 (i.e., *1* − *P* > *P*). Conversely, a positive bias shifts the distribution to the right, enhancing the probability of generating a “1” bit, which results in *P* > 0.5 (i.e., 1 − *P* < *P*). This behavior is experimentally validated by the measurements presented in Fig. 2C, which illustrates the progressive upward shift of the current data points as the bias sweeps across the negative and positive steps. Furthermore, the mapping of the generated p-bits (Fig. 2D) confirms this controllability; by modulating the applied bias, the proportion of “1” bits in the output stream was adjusted from 0% at −300 μV to 100% at +300 μV, and detailed p-bit mappings as a function of bias are provided in fig. S2.

A distinguishing feature of the proposed device is the bias invariant SD (σ) of the Gaussian distributed current signals. In conventional implementations that use thermal or circuit noise, σ changes with bias, which imposes a fundamental limit on quantitative, bias-only control of p-bits. By contrast, our device exhibits a rigid, bias-driven lateral shift of the Gaussian distribution with σ held constant. In particular, the Gaussian distribution shows a small relative SD of about 2.3%, maintaining an average value of 2.48 × 10^−9^ for σ in all control regions (Fig. 2E). This shape preserving mean shift at fixed σ is essential for threshold-based p-bit operation because bias-dependent broadening or narrowing of σ undermines linear tuning. The physical origin of this behavior is analyzed in a later section. The sigmoid curve in Fig. 2F plots the p-bit output probability as a function of the applied bias voltage. Consistent with prior studies on p-bit systems, the control input–probability relation exhibits a stable sigmoid profile. In our device, as the bias becomes more negative, the probability approaches 0%, whereas more positive bias drives it toward 100%; the slope gradually diminishes in the saturation regions, yielding smooth convergence near both limits. To verify the cycle-to-cycle stability of the device, we performed repeated measurements across all bias range to evaluate the repeatability of the stochastic behavior (fig. S3, A to D). The current outputs maintained consistent bias-dependent controllability across all cycles, and the fitted sigmoid curves showed no observable drift in threshold, confirming stable stochastic operation over repeated operation cycles. We further examined the influence of cycle-to-cycle variation on system operation by performing additional p-computing simulations that explicitly incorporate the experimentally observed fluctuations in the bias-probability characteristics. Two types of variations were considered: (i) gradual characteristic changes accumulated over repeated operation and (ii) instantaneous cycle-to-cycle fluctuations. We first evaluated the impact of gradual characteristic changes that may accumulate over repeated operation by performing p-computing simulations that incorporate variations accumulated up to 500 cycles. The experimentally extracted SDs of the bias-probability curves for each p-bit were sequentially accumulated to construct cycle-dependent probability transfer characteristics. As shown in fig. S3 (E to I), even when cycle-to-cycle variation accumulated over 500 cycles was considered, the computational results of the p-computing system remained qualitatively unchanged, indicating stable operation under accumulated variation. The results obtained at intermediate accumulation levels (e.g., 100, 200, 300, and 400 cycles) also exhibited the same qualitative behavior, indicating stable operation across the entire range of accumulated variation.

We next evaluated the impact of instantaneous cycle-to-cycle variation on the behavior of p-computing. The measurement data were divided into several cycle windows (1 to 5, 100 to 105, 200 to 205, 300 to 305, 400 to 405, and 495 to 500 cycles), and the mean and SD of the bias-probability characteristics were extracted for each window. Using these cycle-dependent characteristics, probability transfer curves were constructed for each cycle window, allowing the simulations to reflect variations occurring at different stages of repeated operation. The results indicate that, although cycle-to-cycle variation is present, the average probability transfer characteristics remain stable across cycles. When p-computing simulations were performed using these cycle-dependent bias-probability characteristics, the success probability showed some variation depending on the cycle window. In all cases, only the optimal configuration exceeded the pass-value threshold (0.2), while nonoptimal configurations did not. The optimal solution was therefore consistently selected (fig. S4). We further verified the reproducibility of the proposed device by fabricating 10 additional samples under identical process conditions (fig. S5). All devices exhibited bias-dependent probabilistic behavior, confirming structural uniformity and process consistency.

To elucidate the physical origin of the previously observed probabilistic response and bias controlled characteristics, we analyzed the bias dependence of the photoinduced current in the multiheterojunction structure from a band diagram perspective. To construct the band diagram, optical absorption spectroscopy and ultraviolet photoelectron spectroscopy (UPS) were performed. The optical bandgaps (*E*_g_) of the F8BT and SnO_2_ QDs films were determined using Tauc plots, as shown in fig. S6. The F8BT thin film exhibited a bandgap of 2.40 eV (fig. S6, A and B), while the SnO_2_ QDs film showed a direct optical transition with a bandgap of 4.34 eV (fig. S6, C and D). UPS was performed to examine the valence electronic structure and work function (Φ) of each material. For the F8BT thin film, the work function was measured to be 3.07 eV, and the energy difference between the Fermi level and the valence-band maximum (*E*_F_ − *E*_VBM_) was found to be 1.40 eV (fig. S7, A and B), placing the Fermi level near the middle of the bandgap. In the case of the SnO_2_ QD film, the work function was 3.92 eV, and the *E*_F_ − *E*_VBM_ value was 3.86 eV (fig. S7, C and D), indicating that the Fermi level lies close to the conduction band edge, in agreement with its n-type semiconducting behavior. By combining above characterization results, quantitative energy level estimations for both materials were achieved. The *E*_F_ of each material was taken directly from the measured work function values (*E*_F_ = −Φ, referenced to vacuum level), while the valence band maximum (VBM) was calculated by subtracting *E*_F_ − *E*_VBM_ from the work function. The conduction band minimum (CBM) was then obtained by adding the optical bandgap (*E*_g_) to the VBM. Based on this approach, the energy levels of F8BT were estimated as follows: *E*_F_ = −3.07 eV, VBM = −4.47 eV, and CBM = −2.07 eV. For SnO_2_, the corresponding values were *E*_F_ = −3.92 eV, VBM = −7.78 eV, and CBM = −3.44 eV. These extracted values clearly illustrate the relative band alignment across the heterojunction interface and support the formation of a type II energy level configuration (*40*) that facilitates efficient charge separation in the device (fig. S8). Then, we further characterized the arc-discharge illumination used in the DIPS module. Wavelength-resolved measurements confirmed broadband emission with intrinsic temporal fluctuations that follow an approximately Gaussian distribution (fig. S9), demonstrating it as a reliable stochastic optical input.

Using the band diagram defined above, we first explain the origin and mechanism of p-bit generation under unbiased conditions. Owing to its internal multiheterojunction structure, the device adopts a back-to-back diode configuration, consisting of a pn junction photodiode and a ms junction photodiode arranged in opposition. Within this back-to-back architecture, the wavelength-dependent photoresponse separates into two main ranges, consistent with the optical absorption results described above (fig. S6). At the upper PEDOT:PSS/SnO_2_ QDs ms junction, a response originating from the SnO_2_ QDs appears in the deep ultraviolet region (fig. S6C). At the lower SnO_2_ QDs/F8BT pn junction, the broad visible to ultraviolet (UV) response originates from F8BT (fig. S6A). When a DIPS containing a randomly mixed distribution of wavelengths and intensities is incident through the upper PEDOT:PSS transparent electrode, the ms junction first absorbs the deep ultraviolet component and transmits the remaining wavelengths to the lower pn junction. Consequently, a deep ultraviolet-driven photocurrent response arises at the ms junction, while the pn junction produces a photocurrent response from the remaining spectral components. Both photocurrents essentially originate from the photovoltaic effect (*41*–*43*). The resulting photocurrent flows toward the opposing junctions due to the geometry of the two photodiodes. This behavior can be explained from a band diagram perspective, as shown in fig. S10. In the band diagram, the pn and ms junctions face each other; photogenerated electron-hole pairs are separated by the built-in potentials of both junctions and are driven in opposite, facing directions. Accordingly, the measured current corresponds to the sum of two counterflow photocurrents. In this back-to-back diode, the net current depends on the mixed DIPS illumination, whose wavelength composition and intensity change randomly from one exposure to the next; therefore, three current conditions can be considered. First, due to the difference in light intensity between the deep ultraviolet and the remaining wavelengths, when the photocurrent at the ms junction exceeds that at the pn junction, the sum of the two photocurrents yields a positive net current. However, when the ms junction photocurrent is smaller than that at the pn junction (i.e., the pn junction photocurrent is larger), the net current is negative. If the photocurrent in both directions are the equal, then the photocurrent of the two different directions will cancel each other out, resulting the device exhibiting a low dark current level. Because these three probabilistic cases occur randomly over time, the distribution of current levels shown in Fig. 2C is obtained. These distributions can be converted into p-bits.

To further verify the preceding explanation, we reproduced the described scenarios using two separate light-emitting diode (LED) light sources, as shown in fig. S11. A 265-nm LED was used to selectively activate the ms junction, while a 455-nm LED was used to activate the pn junction. We adjusted the intensities of the two light sources carefully to create a condition in which the photocurrents generated at the pn and ms junctions were of the same magnitude. Under these conditions, as shown in fig. S11A, irradiation at 265 nm resulted in a positive photocurrent from the ms junction. In contrast, when the 455-nm light was applied, a negative photocurrent was observed from the pn junction. Furthermore, when both light sources were applied simultaneously, the resulting currents cancelled each other, as previously described. In addition, as shown in fig. S11B, we examined the case in which the two light sources were applied simultaneously with different intensities, resulting in photocurrents of unequal magnitude at the ms and pn junctions. Under these conditions, the junction exposed to the stronger illumination generated a larger photocurrent. The smaller photocurrent produced at the opposing ms junction partially cancelled out this current, resulting in a net current corresponding to the difference between the initial two currents.

**Fig. 3.**
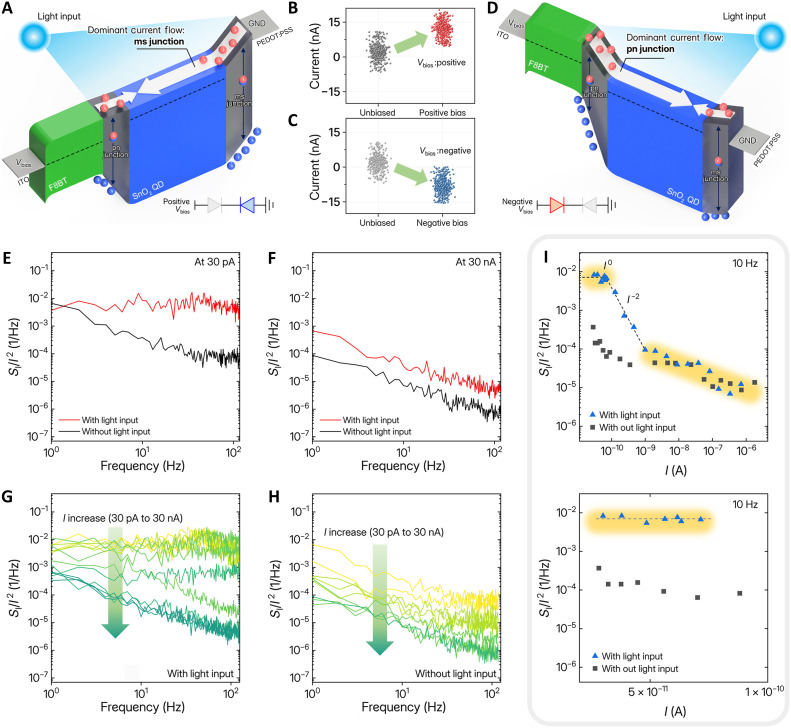
Energy band structure and LFN analysis of the LBP-bit generator under various bias conditions. (**A**) Schematic band diagram illustrating the bending of energy levels in the multiheterojunction structure under a positive bias condition, where GND denotes ground. (**B**) Shift in the distribution of p-bit output in response to an applied positive voltage bias. (**C**) Variation in p-bit output induced by a negative voltage bias. (**D**) The band diagram shows the modulation of the energy bands in the multiheterojunction structure under negative bias conditions. (**E**) LFN spectrum under a low bias current of 30 pA. (**F**) LFN spectrum under a higher bias current of 30 nA. (**G**) Changes in shot noise–dominated noise spectra under illumination as the bias current increases from 30 pA to 30 nA. (**H**) Bias current–dependent behavior of 1/*f* noise under dark conditions. (**I**) Bias current–dependent transition between shot noise and 1/*f* noise at a fixed frequency of 10 Hz.

This behavior represents a unique characteristic of the proposed LBP-bit generator, and we next consider how these dynamic changes under the application of an additional bias. When a bias is applied to the device, energy band bending occurs and influences the direction of the resulting current. Specifically, when a positive voltage bias is applied to the bottom ITO electrode, as shown in Fig. 3A, the pn junction becomes forward biased, reducing its built-in potential, while the ms junction becomes reverse biased, increasing the potential difference and thereby enhancing the internal electric field. As a result, the increased internal electric field more effectively separates the photogenerated electron-hole pairs at the ms junction, leading to a stronger photocurrent. In contrast, the reduced built-in potential at the pn junction results in a weaker photocurrent. In this case, the current from the ms junction becomes dominant within the back-to-back diode structure, and the mean of the resulting p-bit Gaussian distribution shifts toward the positive direction (Fig. 3B). On the other hand, when a negative bias is applied to the device, the behavior becomes the reverse counterpart of the previous case, as illustrated in Fig. 3 (C and D). The resulting energy band deformation causes the pn junction to become reverse biased, enhancing its internal electric field, while the ms junction becomes forward biased, thereby reducing its built-in potential. As a result, and in contrast to the previous condition, a larger photocurrent flows through the pn junction, whereas a weaker current is generated at the ms junction, as shown in Fig. 3D. Consequently, the pn junction becomes the dominant current path within the back-to-back diode structure, and the mean of the resulting p-bit Gaussian distribution shifts toward the negative direction (Fig. 3C).

Furthermore, we conducted low-frequency noise (LFN) analysis (*44*) to gain insight into the bias-independent shift behavior of the Gaussian distribution observed during p-bit control under DIPS illumination. This LFN analysis enables the differentiation between the signal generated by the plasma-driven stochastic optical input within the multiheterojunction structure and the current (*I*) induced by electrical bias. To ensure clear observation of the device’s intrinsic noise characteristics, such as 1/*f* noise and generation/recombination (*g*-*r*) noise, where the intensity of the light source was reduced to minimize the masking effect of excessive shot noise originating from the photocurrent. Under reduced light intensity, both the p-bit outputs and their controllability with respect to bias were concurrently measured, as presented in fig. S12. Under these measurement conditions, the noise characteristics of the LBP-bit generator were compared in the presence and absence of DIPS illumination (Fig. 3, E and F). At a low bias current of 30 pA, light illumination generates shot noise, whereas, in the absence of light, 1/*f* noise intrinsic to the device was observed. However, at a comparatively higher bias current of 30 nA, the noise induced by the electrical bias current exceeded the shot noise level, resulting in dominant 1/*f* noise behavior. We then progressively increased the bias current from 30 pA to 30 nA and compared the noise characteristics under illumination and in the absence of light, as shown in Fig. 3 (G and H). As a result, under illumination, a progressive increase in bias current shows that the shot noise–like behavior remains dominant below a bias of 10-nA level, exhibiting no dependence on the bias magnitude. However, when the bias current exceeds 10 nA, the device becomes dominated by bias-induced 1/*f* noise, and the shot noise–like behavior is no longer observed (Fig. 3G).

This observation supports the p-bit controllability demonstrated above. As shown in fig. S12, the bias current remained below 10-nA level across all applied voltage conditions. Accordingly, in this operational region, the p-bit output fluctuations are governed by the plasma-driven stochastic DIPS illumination and show shot noise–like behavior in the measured current-noise spectrum. The applied bias affected only the energy band bending, as shown in Fig. 3 (A and D), while the resulting low bias current did not introduce a measurable change in the normalized noise spectrum in this operating regime. This indicates that p-bit generation was governed entirely by the photocurrent, with the bias affecting only the band structure. By contrast, in the absence of illumination (Fig. 3H), the 1/*f* noise exhibited a clear dependence on the bias current, with the noise magnitude changing with increasing current. Based on this, Fig. 3I compares noise levels at a fixed frequency as a function of current in both illuminated and nonilluminated conditions, with various levels of bias applied. The results show that, under illumination, the noise level remains the same as the current increase up to 10-nA level. Bias-induced noise becomes dominant, resulting in a region where the magnitude of the noise begins to change in proportion to *I*^−2^. These results confirm that, under low-current conditions relevant for p-bit operation, the normalized noise spectrum shows shot noise–like behavior and remains constant regardless of the applied bias. This bias independence of the normalized noise underlies bias-independent shift behavior of the Gaussian distribution, supporting that the p-bit statistics are primarily set by the stochastic optical input, while the bias controls the mean shift.

We next examined the computational applicability of the proposed p-bit generation and control system through a model-based evaluation grounded in Boltzmann machine principles. As described earlier, p-computing transforms a given problem into an energy function and solves it by identifying the global minimum, following the architecture and operating principles of a Boltzmann machine. In a Boltzmann machine, interactions between binary units are represented using an Ising model, and the probability of each unit’s configuration is described by the Boltzmann distribution, such that lower-energy states occur with higher probability (*45*). P-computing applies the same principle: It defines the energy so that the correct solution corresponds to the global minimum of this function and then repeatedly samples p-bits and takes the configuration that appears most frequently over many samples as the answer. The energy function used in p-computing is defined in the following form

$$H=-\sum_{j\neq i}w_{\mathrm{ij}}x_{i}x_{j}+\sum_{j}\theta_{j}x_{j}$$ (1)

$$I_{i}=-\frac{\partial H}{\partial x_{i}}=\sum_{j\neq i}w_{\mathrm{ij}}x_{j}+\theta_{i}$$ (2)

In this expression, $x_{i}$ and $x_{j}$ denote the outputs of p-bits, $w_{\mathrm{ij}}$ represents the connection strength between two p-bits, and $\theta_{j}$ is the applied bias. The input function ($I_{i}$) for the *i*th p-bit (defined as the partial derivative of the energy with respect to its output) is applied as the p-bit’s input, thus modulating its output probability in the direction of decreasing energy. If p-bits are to update strictly toward lower-energy states, then they become trapped in local minima and fail to reach the global minimum. However, due to their intrinsic stochastic nature, p-bits occasionally update toward higher-energy states, providing the necessary fluctuations to escape local minima and effectively explore the global energy landscape. Thus, p-computing excels at solving complex problems with vast solution spaces and intricate energy landscapes while minimizing the risk of becoming trapped in local minima.

**Fig. 4.**
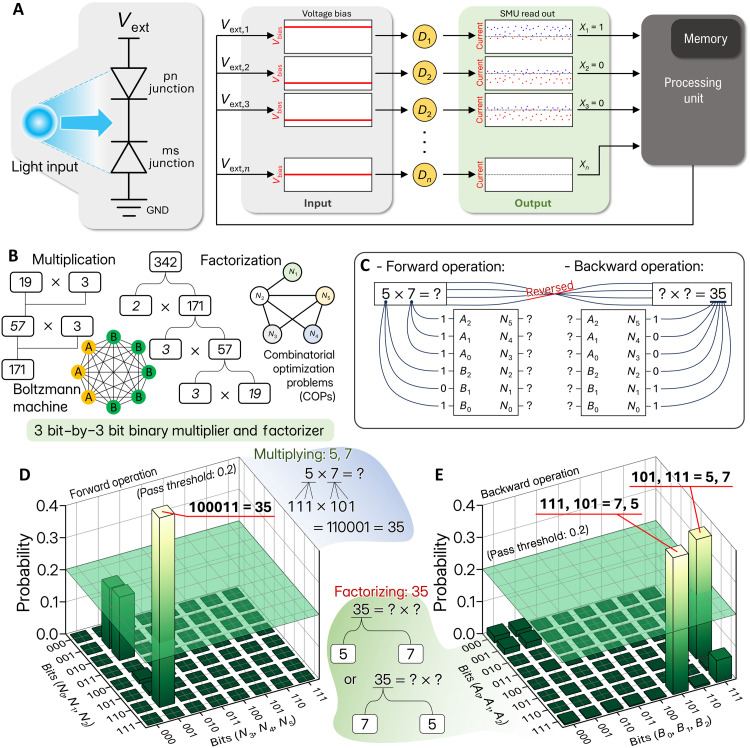
Schematic of p-computing hardware architecture based on the proposed p-bits and its application to integer multiplication and factorization. (**A**) Hardware schematic shows iterative computation of $I_{i}$, conversion to $V_{\text{ext},i}$ and application to each LBP-bit generator. Each p-bit output is sampled stochastically and configurations with probability greater than pass threshold (0.2) are retained. Memory stores sampled configurations over successive iterations. (**B**) Diagram of multiplication and factorization operations based on p-computing. (**C**) Logical mapping of the invertible 3 bit–by–3 bit binary multiplier, where *A* and *B* are encoded as 3-bit factors and *N* as a 6-bit product. Forward operation fixes the factors to determine the product while backward operation fixes the product to find the factor pairs. (**D**) Forward operation results for *A* = 7 (111_2_) and *B* = 5 (101_2_), showing the highest probability for unfixed product, with a maximum at *N* = 35 (100011_2_) exceeding the pass threshold. (**E**) Backward operation results with product fixed to *N* = 35 (100011_2_), showing that the probability of unfixed factors yield two solutions (7, 5) and (5, 7), both exceeding the pass threshold.

Figure 4A illustrates the p-computing hardware architecture based on proposed p-bits. First, all p-bit outputs are initialized randomly. The process unit then computes each p-bit’s $I_{i}$ from this randomly generated configuration. Next, $I_{i}$ is applied to the *i*th p-bit as an external voltage ($V_{\text{ext},i}$), steering the p-bit’s output probability toward lower-energy states. At this stage, $V_{\text{ext},i}$, applied to the p-bit and the resulting p-bit output $x_{i}$ can be written as follows

$$V_{\text{ext},i}=V_{o}+V_{s}I_{i}$$ (3)

$$x_{i}=u\left[\sigma \left(I_{i}\right)-\text{rand}\right]=u\left[\sigma \left(\frac{V_{\text{ext},i}-V_{o}}{V_{s}}\right)-\text{rand}\right]$$ (4)

$V_{o}$ is the offset voltage that yields a 50% output probability for the p-bit and $V_{s}$ is the scaling voltage. These parameters convert the input function $I_{i}$ into the $V_{\text{ext},i}$ and are determined by the sigmoidal fitting curve shown in Fig. 2F. It is noted that *u* denotes the unit step function used to binarize the analog current, and “rand” represents a uniformly distributed random number in [0, 1] that introduces the intrinsic stochasticity of the p-bit during this binarization step. When the process unit applies $V_{\text{ext},i}$ to each p-bit, the LBP-bit generator produces a stochastic current. If this current exceeds a predefined threshold, then the p-bit outputs a “1”; otherwise, it is defined as either “0” or “1.” After sampling the p-bits, the process unit recomputes each p-bit’s $I_{i}$ and repeats the above steps for a fixed number of iterations. Upon completion, the memory containing the p-bit configurations from every iteration is scanned to identify those configurations whose empirical probability exceeds a certain pass threshold (0.2 in this work), and these configurations are selected as the final solution.

This p-computing architecture, which exploits the intrinsic randomness of p-bit devices to avoid trapping in local minima and efficiently locate global minima, shows promise for integer factorization: a problem known to be intractable on classical computers. Factorization is the inverse operation of multiplication, as shown in Fig. 4B. Factorization cannot be solved in polynomial time on classical computers as it requires evaluating numerous possible cases to find the solution. The general number field sieve, the most efficient classical factorization algorithm, exhibits subexponential time complexity, reflecting the inherent combinatorial optimization nature of factorization, which can be efficiently modeled through energy-based probabilistic frameworks such as p-computing (*24*). Figure 4C shows how a 3 bit–by–3 bit binary multiplier’s forward operation (multiplication) and its inverse (factorization) are implemented using invertible logic operations based on p-computing. For all p-computing simulations, the experimentally obtained bias-probability characteristics were refined through sigmoid fitting to mathematically represent the measured probability distributions with continuity for modeling purposes. The two factors (*A* and *B*) are each encoded as 3-bit binary numbers, while the product (*N*) is encoded as a 6-bit binary number. Each bit is mapped to a separate p-bit, for a total of 12 p-bits. Because energy must reach its minimum precisely when the product of *A* and *B* equals *N*, the energy function can be defined as follows

$$H={\left(N-\mathrm{AB}\right)}^{2}$$ (5)

In table S1, $I_{i}$ for all 12 p-bits are summarized, where these values are obtained by taking the partial derivatives of the above energy function. The p-computing network defined by Eq. 5 and table S1 converges to the configuration satisfying *N* = *A* × *B*. Fixing the outputs of the 6 p-bits representing the two factors (*A* and *B*) yields the multiplication *A* × *B* (forward operation), while fixing the outputs of the 6 p-bits representing product (*N*), yields the two factors of *N* (backward operation).

Figure 4D shows the simulation results for 5 × 7 multiplication and 35 factorization, based on the sigmoidal output characteristic of the p-bit measured in Fig. 2F. In the forward operation, the 6 p-bits corresponding to the two factors (*A* and *B*) were fixed to *A*_2_*A*_1_*A*_0(2)_ = 111_(2)_ and *B*_2_*B*_1_*B*_0(2)_ = 101_(2)_ (i.e., 7 and 5). Figure S13A shows the energy of each configuration of the unfixed 6 p-bits corresponding to the product (*N*), where the energy reaches its minimum for *N* = 35. In this forward operation, only the correct product *N*_5_*N*_4_*N*_3_*N*_2_*N*_1_*N*_0(2)_ = 100011_(2)_ exceeded the pass threshold of 0.2, with a probability of 0.37, while all incorrect configurations remained below 0.2. In the backward operation, the 6 p-bits corresponding to the product (*N*) were fixed to *N*_5_*N*_4_*N*_3_*N*_2_*N*_1_*N*_0(2)_ = 100011_(2)_. Figure S13B shows the energy of each configuration of the unfixed 6 p-bits corresponding to the two factors (*A*, *B*), with the minimum energy observed for (*A*, *B*) = (5, 7) or (7, 5). In this backward operation, only the correct configurations, where *A*_2_*A*_1_*A*_0(2)_ = 111_(2)_ and *B*_2_*B*_1_*B*_0(2)_ = 101_(2)_ and *A*_2_*A*_1_*A*_0 (2)_ = 101_(2)_ and *B*_2_*B*_1_*B*_0(2)_ = 111_(2)_, exceeded the pass threshold of 0.2, with probabilities of 0.40 and 0.41, respectively, while all incorrect configurations remained below 0.2. Therefore, 35 was successfully factorized with a solution accuracy of 0.81.

To further expand into the invertible logic capability of the proposed system, we implemented a 3–p-bit network for an AND gate with two inputs (*X* and *Y*) and one output (*Z*) (*16*, *27*, *30*), as shown in fig. S14. The system successfully executed both forward and backward operations based on the energy function $H={\left(Z-\mathrm{XY}\right)}^{2}$ validating its applicability to inversible Boolean logic. In the forward operation (fixing *X* and *Y* to compute *Z*), the probability of producing the correct truth-table outcome exceeded 0.8, confirming high accuracy. In the backward operation (fixing *Z* to find *X* and *Y*), only valid input combinations exceeded the pass threshold of 0.2. By defining energy functions in this manner for any truth table, diverse invertible logic gates can be realized and concatenated to implement more complex logic operations.

**Fig. 5.**
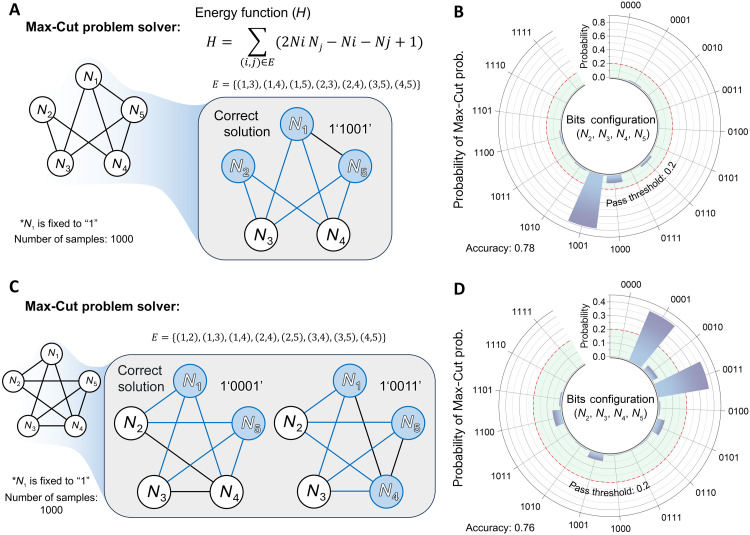
Demonstration of p-computing for solving Max-Cut problems. (**A**) five-node seven-edge graph used for the first Max-Cut instance. *N*_1_ is clamped to 1 to remove global bit flip degeneracy. (**B**) Radial histogram over the remaining bits (*N*_2_, *N*_3_, *N*_4_, and *N*_5_) showing the most frequently counted case corresponding to the correct cut. The solution accuracy (fraction of trials yielding the correct cut) is 0.78. (**C**) Five-node eight-edge graph for the second Max-Cut instance with the same fixed *N*_1_. (**D**) Radial histogram for the second instance showing two dominant configurations. Both correct cuts exceed the pass threshold, and the solution accuracy is 0.76.

We next explored the applicability of our system to more complex combinatorial optimization problems (COPs) (*46*–*48*). While COPs have a finite set of possible solutions, the size of the solution space increases exponentially with problem size, making exhaustive searches on classical computers practically infeasible. P-computing uses the intrinsic randomness of its hardware to escape local minima and efficiently explore a vast solution space in pursuit of the global minimum, offering a clear advantage for solving COPs. The Max-Cut problem is a canonical COP, with active applications in VLSI circuit design, social network analysis, and logistics optimization (*49*). In the Max-Cut problem, given a graph consisting of vertices and edges, the objective is to partition the vertices into two subsets such that the number of edges (the cut value) connecting vertices in different subsets is maximized. By mapping each vertex to a p-bit and defining the partition based on the p-bit outputs, the energy function can be expressed as follows

$$H=\sum_{\left(i,j\right)\in E}\left(2N_{i}N_{j}-N_{i}-N_{j}+1\right)$$ (6)

where $N_{i}$ and $N_{j}$ denote the outputs of the p-bits corresponding to vertices *i* and *j* and E is the set of edges connecting vertex pairs. According to Eq. 6, a neighboring pair in state (1, 1) or (0, 0) incurs an energy increment of 1, whereas (1, 0) or (0, 1) does not. Therefore, the configuration that maximizes the cut value corresponds to the global energy minimum. Figure 5 shows two distinct five-node Max-Cut instances and their simulated solutions. The energy functions and corresponding input functions for these instances are summarized in figs. S15 and S16 and tables S2 and S3, where the energy of every unfixed p-bit configuration is also plotted. Because the cut value is invariant under a global bit flip, we fixed $N_{1}=1$ to reduce the solution space. In fig. S15 (associated with Fig. 5A), the energy landscape exhibits a unique global minimum, whereas, in fig. S16 (associated with Fig. 5C), there are two. Figure 5 (B and D) demonstrates that only the configurations at these global minima achieved probabilities above the pass threshold of 0.2. To verify that this property persists over multiple stochastic operations, we repeated the p-computing procedure 100 times for each Max-Cut instance. As shown in fig. S17, the 100-trial averages reproduced the single-run behavior: the instance corresponding to Fig. 5A yielded an average accuracy value of 0.73, and the instance corresponding to Fig. 5C yielded 0.76 (both clearly above the pass threshold of 0.2) presenting the same trend under repeated operation. Additional simulations on two more five-node Max-Cut problems, with energy and input functions in tables S4 and S5 and results in fig. S18, correctly identified all valid cuts. Consequently, the four instances tested yielded an average solution accuracy of 0.72, demonstrating that the LBP-bit generator–based p-computing architecture can function as a general-purpose Max-Cut solver. To further examine the scalability of the proposed system, we performed additional p-computing simulations on larger Max-Cut problems. In addition to the previously presented five-node case, we considered larger problem instances with 15 and 20 nodes and carried out computations using the experimentally characterized probability transfer function, including its associated uncertainty. As shown in fig. S19, the expanded simulations yielded an average accuracy of 0.81 for the 15-node problem and 0.72 for the 20-node problem. In both cases, the probability of the optimal solution consistently exceeded the pass-value threshold (0.2). Furthermore, when the computations were repeated 100 times under each condition, the uncertainty bars shown in fig. S19 indicate that the variation of the optimal solution never dropped below the threshold, while the variation of nonoptimal solutions did not exceed it. These results show that, even as the number of possible configurations increases substantially, from 25 in the five-node case to 215 and 220, the optimal solution can still be reliably identified under conditions that include uncertainty in the probability transfer characteristics and cycle-to-cycle variation. This observation suggests that the small-scale demonstrations presented in this work are not merely illustrative examples, but that the probabilistic operation of the proposed device remains valid for more complex problem settings. We also repeated the experiments with the sigmoid curve from fig. S12B, in addition to the curve used previously, and carried out the forward and inverse multiplier operations as well as the Max-Cut computation; the corresponding results are summarized in fig. S20.

To clarify the functional role of a shape-preserving mean shift at a fixed σ for threshold-based p-bit operation, we further analyzed the probabilistic behavior of our device. In a thresholded Gaussian-noise p-bit, the output probability is defined by the likelihood that the noisy signal exceeds a fixed threshold. When the mean of the underlying noise distribution shifts while σ remains unchanged, the resulting exceedance probability follows an analytical sigmoid function. Because this sigmoid is symmetric around *P* = 50%, equal positive and negative bias perturbations lead to equal and opposite probability variations, enabling linear, reversible, and quantitatively predictable control. Such control is essential for mapping the input function (*I*_i_) of a Boltzmann machine into the p-bit’s probability domain.

If σ instead increases with the applied bias, then the probability-bias relationship deviates from the ideal sigmoid form. Broadening of σ stretches the distribution tails and disrupts the monotonic and symmetric bias-probability mapping. As a result, the p-bit can no longer represent the intended *I*_i_ accurately, resulting in unstable energy minimization and frequent failure to reach the global minimum. To confirm this effect, we conducted an additional experiment using the same five-node Max-Cut task from Fig. 5C. The experimentally measured data in fig. S21A, which shows a nearly bias-independent σ, were extracted and used as a reference to construct a hypothetical bias–dependent σ model for the simulations in fig. S21 (B and C). When this σ-bias coupling was introduced, the correct solutions in fig. S21 (E and F) only marginally exceeded or failed to exceed the pass threshold, resulting in solver accuracies of 47.5 and 35.1%. In contrast, our device maintains a nearly constant σ, which preserves an ideal sigmoid and ensures stable probabilistic tuning for reliable p-computing operation.

## DISCUSSION

In summary, this work presented an approach to physical p-bit generation by photoinduced charge polarity as a source of controllable entropy. In the proposed p-bit device architecture, the separation of randomness generation and probability tuning enables submillivolt control with a bias three orders of magnitude lower than previous reports while achieving lower power consumption and preserving signal shape and stability (Table 1). This decoupling simplifies circuit complexity and offers a scalable, energy-efficient route toward practical p-computing.

**Table 1.** Comparison of p-bit implementations. MTJ, magnetic tunnel junction; NA, not applicable.

Device type	Source of stochastic	Probability control function	Probability generating function	Power consumption	Demonstration and application	Year	Ref.
MTJ	Spin-transfer-torque switching	Current, −0.3 to 0.3 mA		260 pJ/bit	NA	2022	(*19*)
Memristor	Reversible metallic filament	Voltage, 2.75 to 2.85 V		NA	NA	2022	(*13*)
Memristor	Reversible ion migration	Voltage, −6 to −3.5 V		NA	Factorization, Boolean SAT	2022	(*15*)
Memristor	Reversible ion migration	Voltage, 4.8 to 5.6 V		154 pJ/bit	Boolean logic, half adder, 2 bit–by–2 bit multiplier	2022	(*27*)
Memristor	Noise-induced self-oscillation	Voltage, 1.32 to 1.46 V		128 pJ/bit	Minimum vertex covering, Max-Cut	2023	(*10*)
MTJ	Spin-orbit-torque switching	Voltage, 0.4 to 1.4 V		NA	Probability distribution function	2024	(*18*)
Bistable resistor	Impact ionization	Voltage, 3.2 to 4.4 V		58 pJ/bit	Max-SAT	2024	(*30*)
Back-to-back diode	Net photocurrent of the multiheterojunction	Voltage, −300 to 300 μV	Light input (DIPS)	65 pJ/bit	Boolean logic, factorization, Max-Cut	2025	This work

With these finely tuned p-bits, we implemented both the forward operation (multiplication) and reverse operation (factorization) of a 3 bit–by–3-bit binary multiplier. Invertible AND gate operation was implemented to demonstrate invertible logic capability of the proposed system. The five-node Max-Cut problem was successfully evaluated on four different graphs, and the approach was further validated on larger problem instances with 15 and 20 nodes, where the optimal solutions were reliably identified despite the increased solution space. These findings show that light-driven p-bits can underpin flexible and energy-efficient p-computing for logic, inference, and decision-making. Such computing system will require continued exploration of materials, device architectures, and algorithmic frameworks that can fully harness the properties of photonic entropy. In this point of view, we imagine computing systems where light becomes more than a signal, where randomness is not just accepted but intentionally designed into the way technologies learn and reason.

## MATERIALS AND METHODS

### Material preparation, nanoparticle synthesis, and device fabrication

#### Reagents

Tin tetrachloride solution (SnCl_4_; 98%, 1.0 M in CH_2_Cl_2_), tert-butanol (≥99.5%), chlorobenzene (≥99.5%), chloroform (≥99%), isopropyl alcohol (IPA; ≥99.9%), anhydrous ethanol (≥99.5%), F8BT (average *M*_w_ > 20,000), and PEDOT:PSS (3 to 4 wt % in H_2_O, high-conductivity grade) were purchased from Sigma-Aldrich and used as received. ITO patterned quartz substrates (15 mm by 15 mm, RnD Korea) were sequentially sonicated in deionized water, ethanol, and IPA for 20 min each and then dried with N_2_.

#### Synthesis of SnO_2_ QDs

SnCl_4_ precursor (50 mmol) was introduced into 100 ml of anhydrous tert-butanol under vigorous stirring until a transparent solution formed. The mixture was sealed in a Teflon-lined stainless-steel autoclave and heated at 100°C for 24 hours. After cooling to room temperature, the resulting white colloid was collected by centrifugation, washed three times with acetone, and dried under vacuum, yielding SnO_2_ QDs.

#### Preparation of coating solutions

SnO_2_ QDs were dispersed at 30 mg ml^−1^ in a chloroform/ethanol cosolvent (75:25 v/v) by bath sonication. F8BT was dissolved in chlorobenzene at 7 mg ml^−1^. PEDOT:PSS was diluted with IPA (3:1 v/v) to improve film conductivity and wetting.

#### Device fabrication

On the cleaned ITO/quartz substrates, SnO_2_ QD and F8BT layers were sequentially spin coated at 3000 rpm for 30 s each, followed by baking at 150°C for 1 hour to remove residual solvents and promote film densification. The top electrode was formed by spin coating the PEDOT:PSS/IPA mixture at 2400 rpm for 30 s and annealing under identical conditions. Completed devices were stored under ambient laboratory conditions until measurement.

### Characterization of the devices

#### Optical properties

Optical absorption spectroscopy of individual layers were recorded using a UV-visible spectrophotometer (Lambda 750, PerkinElmer). The electronic energy levels of active materials were probed by ultraviolet photoemission spectroscopy (UPS, AXIS SUPRA, Kratos) at the Korean Basic Science Institute. High-resolution XPS and XAS spectra were acquired at the 10A2 HR-PES II beamline of the Pohang Accelerator Laboratory (PAL, Republic of Korea) using a synchrotron x-ray source (*50*). The excitation energy was calibrated to the gold binding energy at 84 eV with a gold-foil reference, and a pass energy of 20 eV was used for narrow scans.

#### Electrical and optoelectronic measurements

Current-voltage (*I*-*V*) characteristics and time-resolved photocurrent responses were measured with a source meter (Keithley 2400, Tektronix) under illumination from 265 nm (M265 L5, Thorlabs) or 455 nm (M455 L4, Thorlabs) LEDs. For LFN measurements, the current was routed through shielded triaxial cables into a low-noise current preamplifier (SR570, Stanford Research Systems) and biased using a semiconductor parameter analyzer (Keysight B1500A). The amplified voltage signal was then fed into an FFT analyzer (35670A, Agilent) to extract the power spectral density.

### Software simulation

#### Invertible multiplier, AND logic, and Max-Cut solving

The software simulations for the invertible multiplier, AND logic, and Max-Cut solving were performed using Python. The measured sigmoidal curve, which represents the probability modulated by the applied control voltage bias, was used to extract the output voltage, while the input functions were computed from the p-bit outputs in Python. The operation was repeated for the specified number of cycles to obtain the probabilities of each p-bit configuration and identify the solutions (see supplementary text 2 for details).

## Supplementary Materials

### This PDF file includes:

Supplementary Texts 1 and 2 Figs. S1 to S21 Tables S1 to S5
