# Supplementary material for: Sub-mV tunable photonic p-bits for probabilistic computing
Source: Sci Adv. 2026 May 15;12(20):eaeb9277. doi: 10.1126/sciadv.aeb9277 (PMC13178548; doi:10.1126/sciadv.aeb9277)
Supplement: Supplementary file 1 — Supplementary Texts 1 and 2 Figs. S1 to S21 Tables S1 to S5 [file sciadv.aeb9277_sm.pdf]

Science Advances
AAAS

Supplementary Materials for

Sub-mV tunable photonic p-bits for probabilistic computing

Juhyung Seo *et al.*

Corresponding author: Joon-Kyu Han, joonkyuhan@snu.ac.kr; Hocheon Yoo, hocheon@hanyang.ac.kr

Sci. Adv. **12**, eaeb9277 (2026)
DOI: 10.1126/sciadv.aeb9277

This PDF file includes:

Supplementary Texts 1 and 2
Figs. S1 to S21
Tables S1 to S5

Supplementary Text 1.

XPS and XAS characterization of the employed materials.

To confirm whether the synthesized material adopts the intended SnO_2 phase, X-ray photoelectron spectroscopy (XPS) analysis was performed. As shown in fig. S1A, the SnO_2 QD film exhibits distinct peaks corresponding to the constituent elements of SnO_2, including Sn *3d*, Sn *4d*, and O *1s*. Similarly, in the case of F8BT, characteristic peaks of its elemental components (O *1s*, N *1s*, C *1s*, and S *2p*) are clearly observed, as shown in fig. S1B.

The local electronic structure and oxidation state of the synthesized SnO_2 QDs was investigated by using soft X-ray absorption spectroscopy (XAS). The Sn $M_{4,5}$-edge and O K-edge spectra, shown in fig. S1C and S1D respectively, provide insight into the chemical state of Sn and the hybridization environment of oxygen. The Sn M-edge region (fig. S1C) exhibits six distinct features, labeled Feature A through Feature F. Feature A, located near 486 eV, is observed as a low-energy shoulder commonly associated with partially reduced Sn^{2+} species, suggesting the presence of slight oxygen deficiency(51). Features B (~489 eV), C (~492 eV), and D (~495 eV) represent the main absorption peaks of Sn^{4+} arising from 3d to 5p transitions, which are characteristic of stoichiometric SnO_2(51). Features E and F, appearing at higher energies (~499–505 eV), are attributed to additional transitions and multiple scattering processes linked to extended conduction band states(52). The coexistence of Feature A with dominant Sn^{4+} signals implies a minor degree of Sn reduction, likely due to oxygen vacancies. These vacancies act as donor sites, releasing free electrons into the conduction band and thereby contributing to the n-type semiconducting behavior of the material.

The O K-edge spectrum (fig. S1D) shows three main features (Feature G (~531 eV), Feature H (~538 eV), and Feature I (~560 eV)), corresponding to transitions into Sn 5s-bonding, Sn 5p-antibonding, and extended conduction band states, respectively(53). The well-defined nature and relative intensity of these features confirm the formation of an ordered Sn–O bonding network. In particular, the shape of Feature G is consistent with that of crystalline SnO_2.

Supplementary Text 2.

P-Computing Simulation Framework.

The simulation framework has three main components: the experimentally measured p-bit probability function from Fig. 2f, an energy function that encodes the problem, and an iterative sampling loop. The p-bit exhibits a sigmoid probability-bias response between -300 and +300 µV. Energy functions are defined such that their global minimum corresponds to the correct solution. For an AND gate, $H = (Z - XY)^2$, and input functions $I_i = -dH/dx_i$. These input functions convert to bias voltages via sigmoid inverse mapping, then p-bits sample stochastically. Fixing constraints determine which bits are fixed and which evolve. Solutions are configurations appearing with probability above 0.2. For multipliers or max-cut problems, the loop structure remains identical but energy functions become more elaborate.

P-bit Sampling Implementation: P-bit sampling has two steps. First, mu_sampling looks up the probability at a given voltage from the pre-computed data table, samples around it with measured variance, and returns the quantized probability. Second, bit_sampling generates a binary output by comparing this probability to a random number.

(Python code)
```
def mu_sampling(self, v_in):
    row = self.pbit_data.iloc[(self.pbit_data["V_IN"] - v_in).abs().idxmin()]
    sampled_p = np.random.normal(row["P"], row["SD"])
    arr = self.pbit_data["P"].values
    return arr[np.abs(arr - sampled_p).argmin()]

def bit_sampling(self, mu):
    return int(np.random.rand() < mu)
```

Voltage Computation and Fixing Constraints: Input functions convert to bias voltages using the sigmoid inverse mapping. Fixed '0' p-bits get minimum voltage, fixed '1' p-bits get maximum voltage, and free p-bits map continuously based on the input function value.

(Python code)
```
def get_v_in(self, *cost):
    v_in_min = min(self.pbit_data["V_IN"])
    v_in_max = max(self.pbit_data["V_IN"])
    self.V_dict = {key: max(v_in_min, min(round(((cost_value / self.popt[1]) +
self.popt[0]), 3), v_in_max)) for key, cost_value in zip(self.inputs.keys(),
cost)}
    for key, ps in self.pin_status.items():
        if ps == 0: self.V_dict[key] = v_in_min
        elif ps == 1: self.V_dict[key] = v_in_max
        elif ps == 1: self.V_dict[key] = v_in_max
```

AND Gate I_i: The AND gate energy H = (Y1 – X1X2)2 yields Iis that are weighted by input amplitude I0. These functions compute the forces driving each p-bit toward lower energy configurations that satisfy the AND truth table.

(Python code)
```
def and_cost(self):
    I_x1 = self.I_0 * (-self.inputs["x2"] + (2 * self.inputs["x2"] *
self.inputs["y1"]))
    I_x2 = self.I_0 * (-self.inputs["x1"] + (2 * self.inputs["x1"] *
self.inputs["y1"]))
    I_y1 = self.I_0 * ((2 * self.inputs["x1"] * self.inputs["x2"]) - 1)
    return I_x1, I_x2, I_y1
```

Iterative Sampling Loop: The sampling loop initializes p-bits randomly, respecting p-bit constraints. Free p-bits start with random binary values; fixed p-bits (‘0’ or ‘1’) keep their assigned values. The loop then executes for a specified number of iterations. At each iteration: (1) compute input functions from the current configuration using the cost function, (2) convert input functions to voltages with get v_in, (3) for each free p-bit, obtain its probability with mu_sampling and generate a binary bit with bit_sampling, (4) recompute voltages after each bit update to reflect the changed state, (5) store the updated configuration. After all iterations complete, the collected configurations are organized into a pandas DataFrame where each column is one p-bit and each row is one sample. This enables identifying which configurations appear most frequently and which exceed the probability threshold.

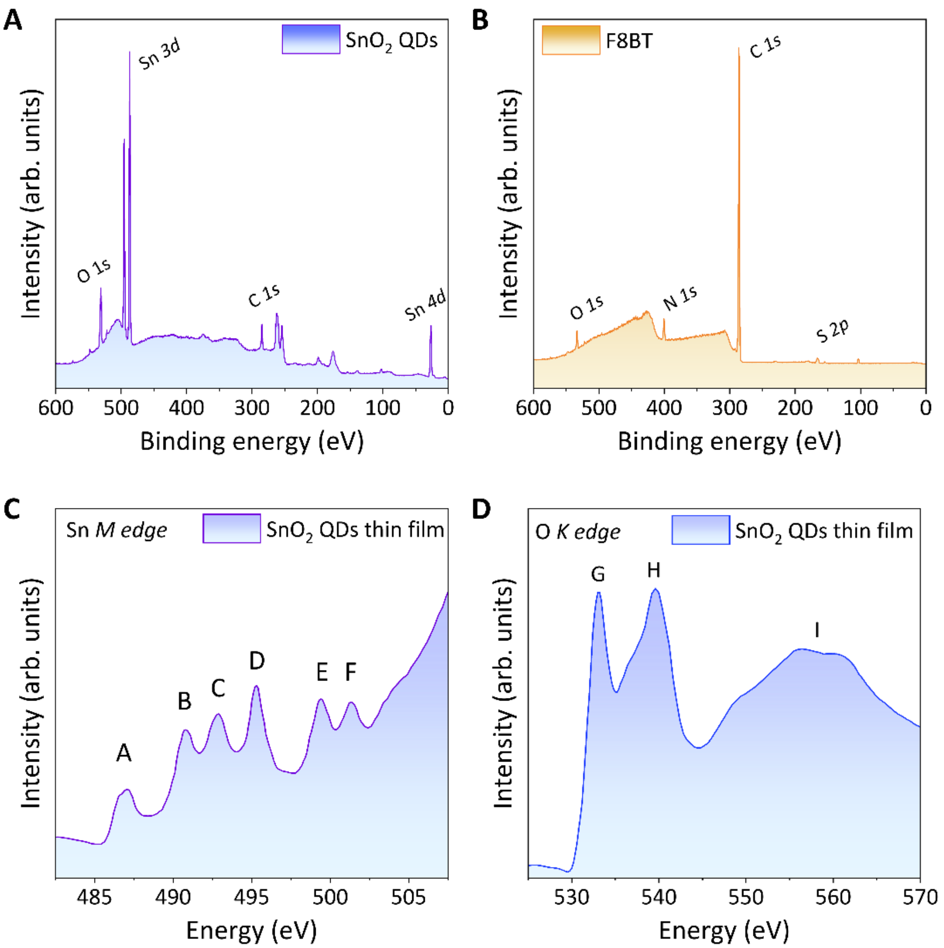

Fig. S1.

X-ray spectroscopic analysis of SnO_2 QDs and F8BT films. (A) XPS survey spectra of SnO_2 QDs, showing characteristic Sn 3d and O 1s peaks. (B) XPS survey spectra of F8BT films, displaying peaks corresponding to C 1s, N 1s, O 1s, and S 2p. (C) Sn $M_{4,5}$-edge XAS spectrum of SnO_2 QDs, indicating the oxidation state and conduction band structure. (D) O K-edge XAS spectrum of SnO_2 QDs, confirming the formation of an ordered Sn–O bonding network.

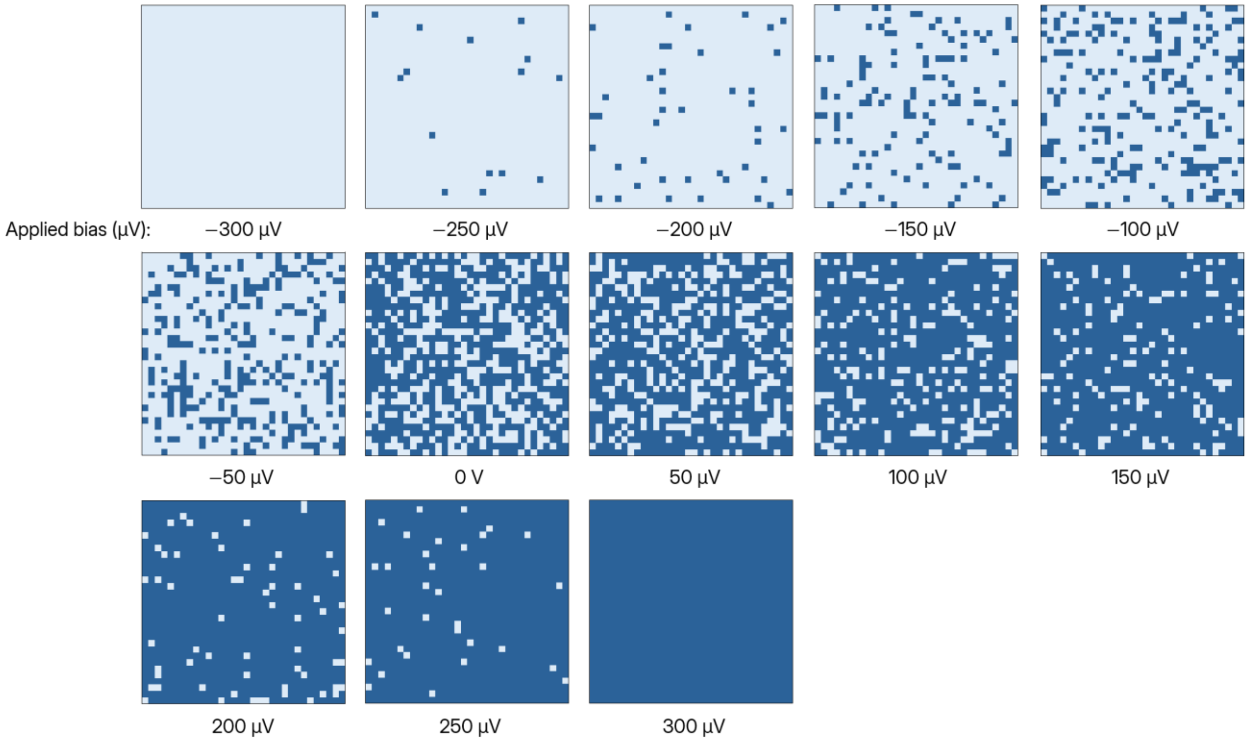

Fig. S2.

Mapping images of p-bits generated under various voltage bias steps. Visualization mapping image of p-bit configurations under incrementally applied bias voltages from –300 μV to 300 μV at 50 μV steps.

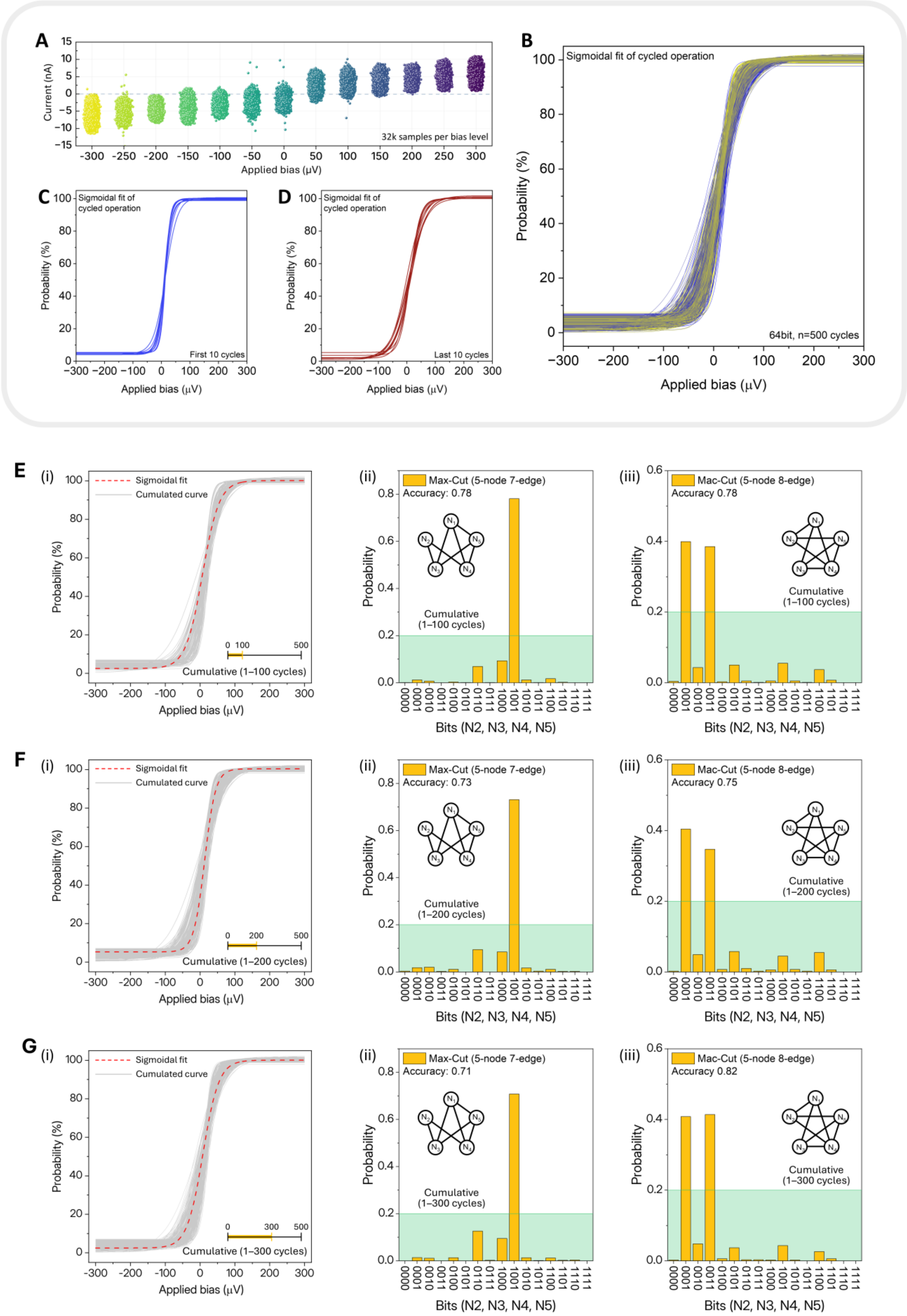

A
Current (nA)
32k samples per bias level
Applied bias (μV)
B
Sigmoidal fit of cycled operation
Probability (%)
64bit, n=500 cycles
Applied bias (μV)
C
Sigmoidal fit of cycled operation
First 10 cycles
Probability (%)
Applied bias (μV)
D
Sigmoidal fit of cycled operation
Last 10 cycles
Probability (%)
Applied bias (μV)
E
(i)
Sigmoidal fit
Cumulated curve
Cumulative (1–100 cycles)
Probability (%)
Applied bias (μV)
(ii)
Max-Cut (5-node 7-edge)
Accuracy: 0.78
Cumulative (1–100 cycles)
Probability
Bits (N2, N3, N4, N5)
(iii)
Mac-Cut (5-node 8-edge)
Accuracy 0.78
Cumulative (1–100 cycles)
Probability
Bits (N2, N3, N4, N5)
F
(i)
Sigmoidal fit
Cumulated curve
Cumulative (1–200 cycles)
Probability (%)
Applied bias (μV)
(ii)
Max-Cut (5-node 7-edge)
Accuracy: 0.73
Cumulative (1–200 cycles)
Probability
Bits (N2, N3, N4, N5)
(iii)
Mac-Cut (5-node 8-edge)
Accuracy 0.75
Cumulative (1–200 cycles)
Probability
Bits (N2, N3, N4, N5)
G
(i)
Sigmoidal fit
Cumulated curve
Cumulative (1–300 cycles)
Probability (%)
Applied bias (μV)
(ii)
Max-Cut (5-node 7-edge)
Accuracy: 0.71
Cumulative (1–300 cycles)
Probability
Bits (N2, N3, N4, N5)
(iii)
Mac-Cut (5-node 8-edge)
Accuracy 0.82
Cumulative (1–300 cycles)
Probability
Bits (N2, N3, N4, N5)

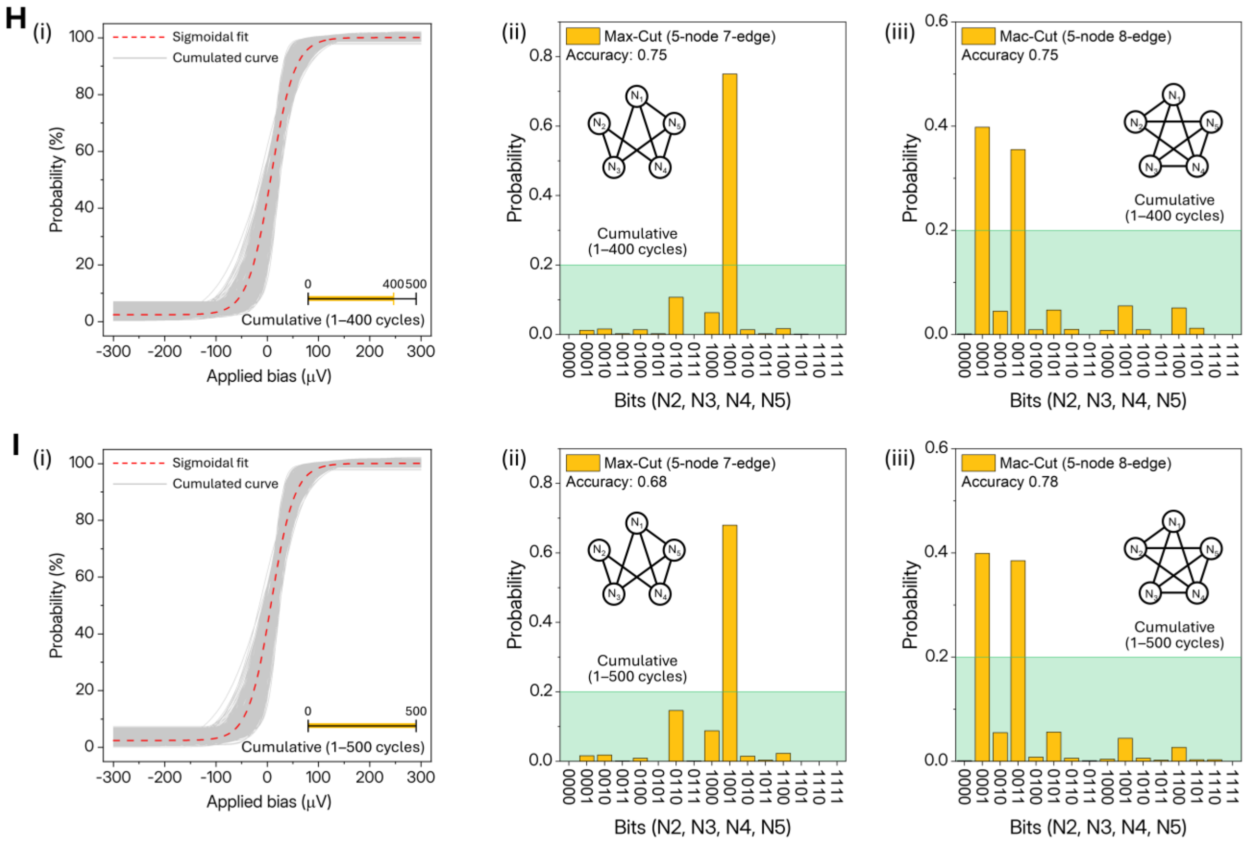

Fig. S3.

Cycle-to-cycle stability of the LBP-bit generator and its impact on probabilistic computing. (A) Raw current outputs measured under repeated cycling, showing consistent bias-dependent controllability across 500 measurement cycles (–300 μV to +300 μV in 50 μV steps). (B) Sigmoid fitting curves of the probability-bias characteristics over 500 cycles, showing no significant variation in slope or threshold and confirming stable probabilistic behavior. Fitted sigmoid curves from the (C) first and (D) last ten cycles. (E-I) Effect of accumulated cycle-to-cycle variation on p-computing performance. For each cumulative cycle range (accumulated in steps of 100 cycles), (i) bias-probability characteristics constructed from the accumulated data, solution probability distribution for the (ii) Max-Cut problem (5-node, 7-edge), and the (iii) Max-Cut problem (5-node, 8-edge) are shown. The results confirm that stable p-computing operation is maintained even as accumulated variation increases up to 500 cycles.

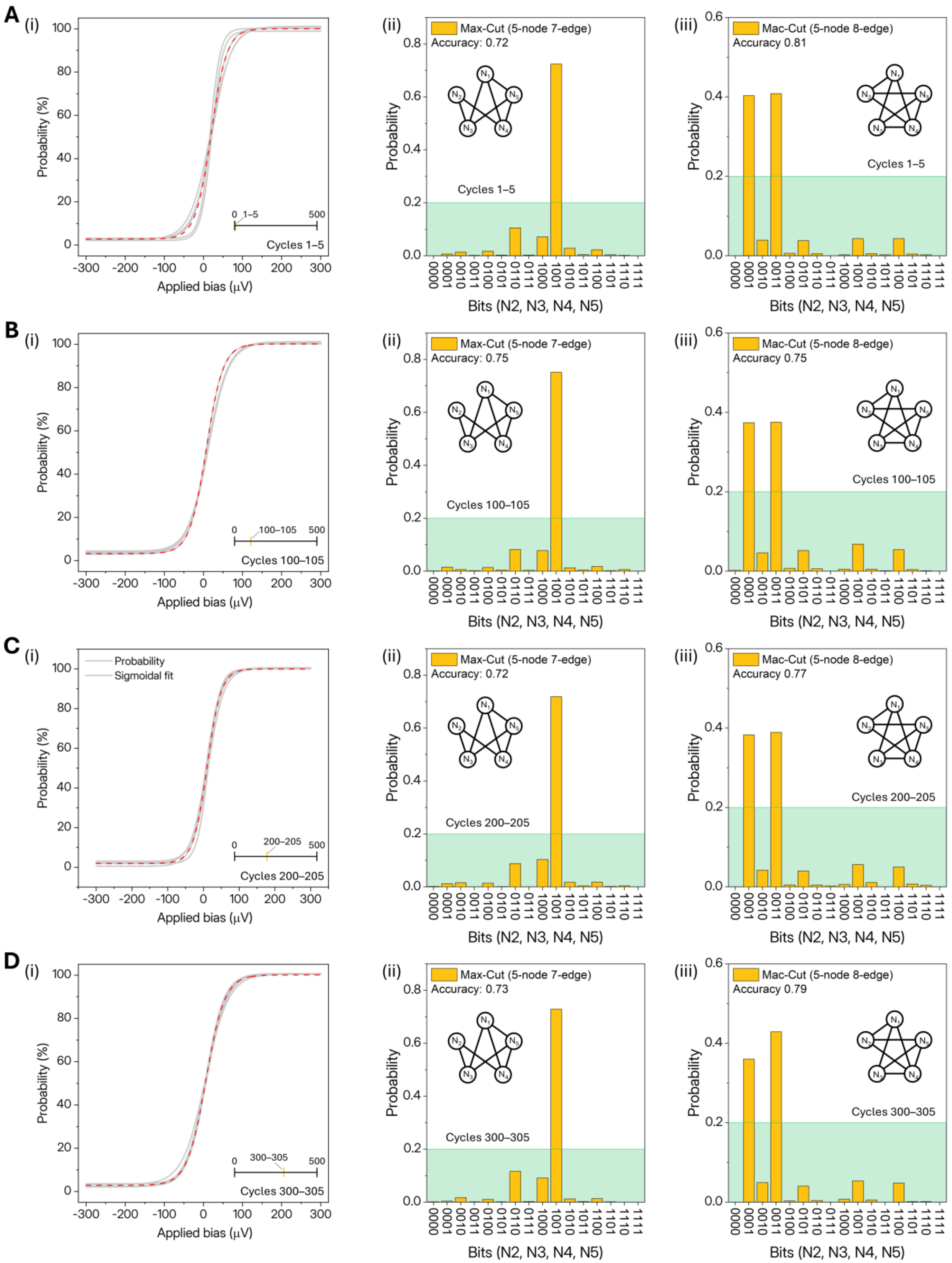

A
B
C
D
(i)
(ii)
(iii)
Probability (%)
Applied bias (μV)
Probability
Sigmoidal fit
0
500
1–5
100–105
200–205
300–305
Cycles 1–5
Cycles 100–105
Cycles 200–205
Cycles 300–305
Max-Cut (5-node 7-edge)
Accuracy: 0.72
Accuracy: 0.75
Accuracy: 0.72
Accuracy: 0.73
Mac-Cut (5-node 8-edge)
Accuracy 0.81
Accuracy 0.75
Accuracy 0.77
Accuracy 0.79
Probability
Bits (N2, N3, N4, N5)
N_1
N_2
N_3
N_4
N_5
0000
0001
0010
0011
0100
0101
0110
0111
1000
1001
1010
1011
1100
1101
1110
1111

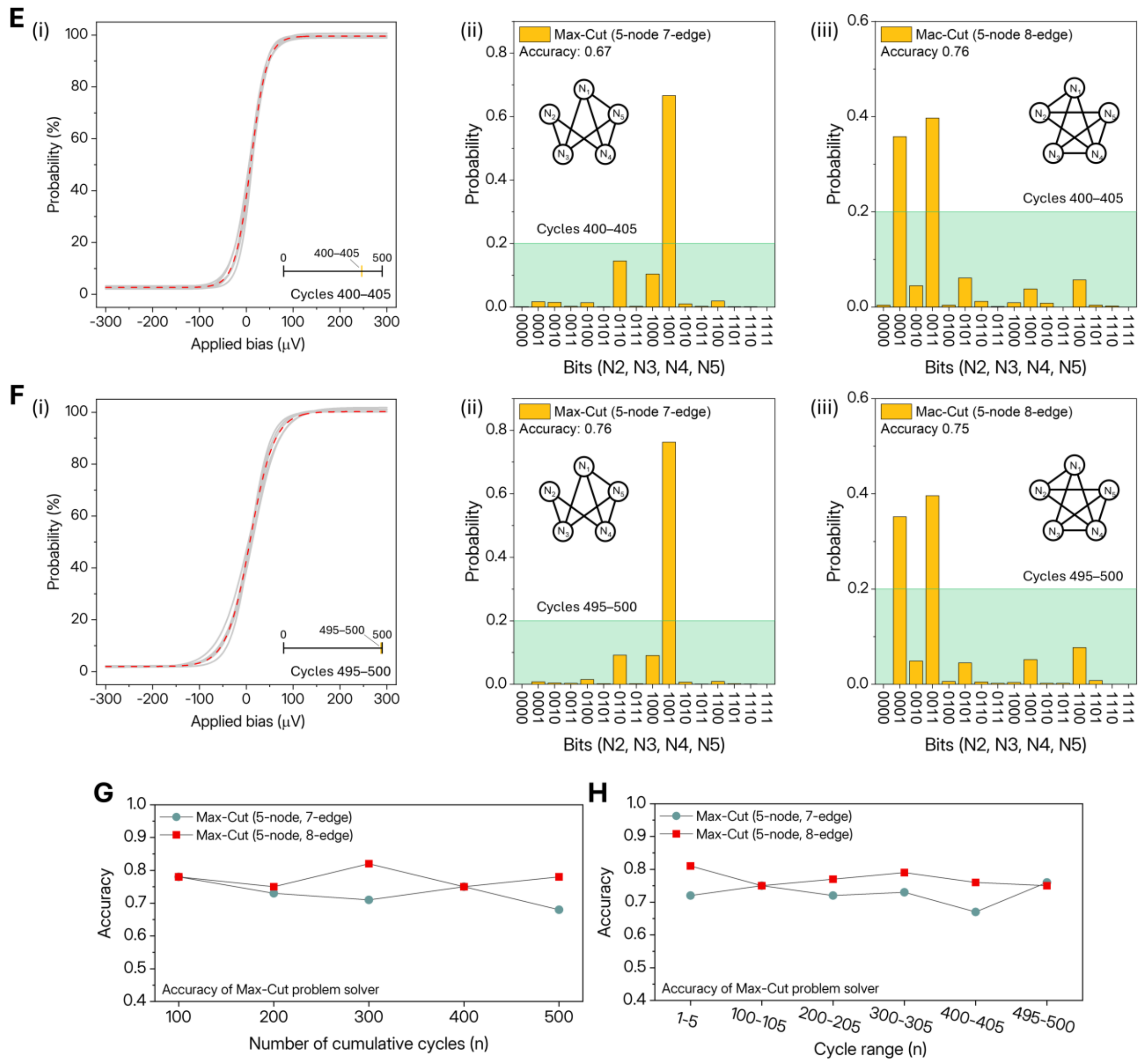

Fig. S4.

Effect of instantaneous cycle-to-cycle variation on p-computing. (A-F) Bias-probability characteristics extracted from different cycle windows (1-5, 100-105, 200-205, 300-305, 400-405, and 495-500 cycles). For each cycle window, (i) bias-probability characteristics and solution probability distributions for the Max-Cut problem with 5 nodes and (ii) 7 or (iii) 8 edges, are shown. (G) Accuracy of the Max-Cut solver as a function of cumulative cycles. (H) Accuracy of the Max-Cut solver as a function of cycle window position. The results confirm that stable p-computing operation is maintained across different cycle windows.

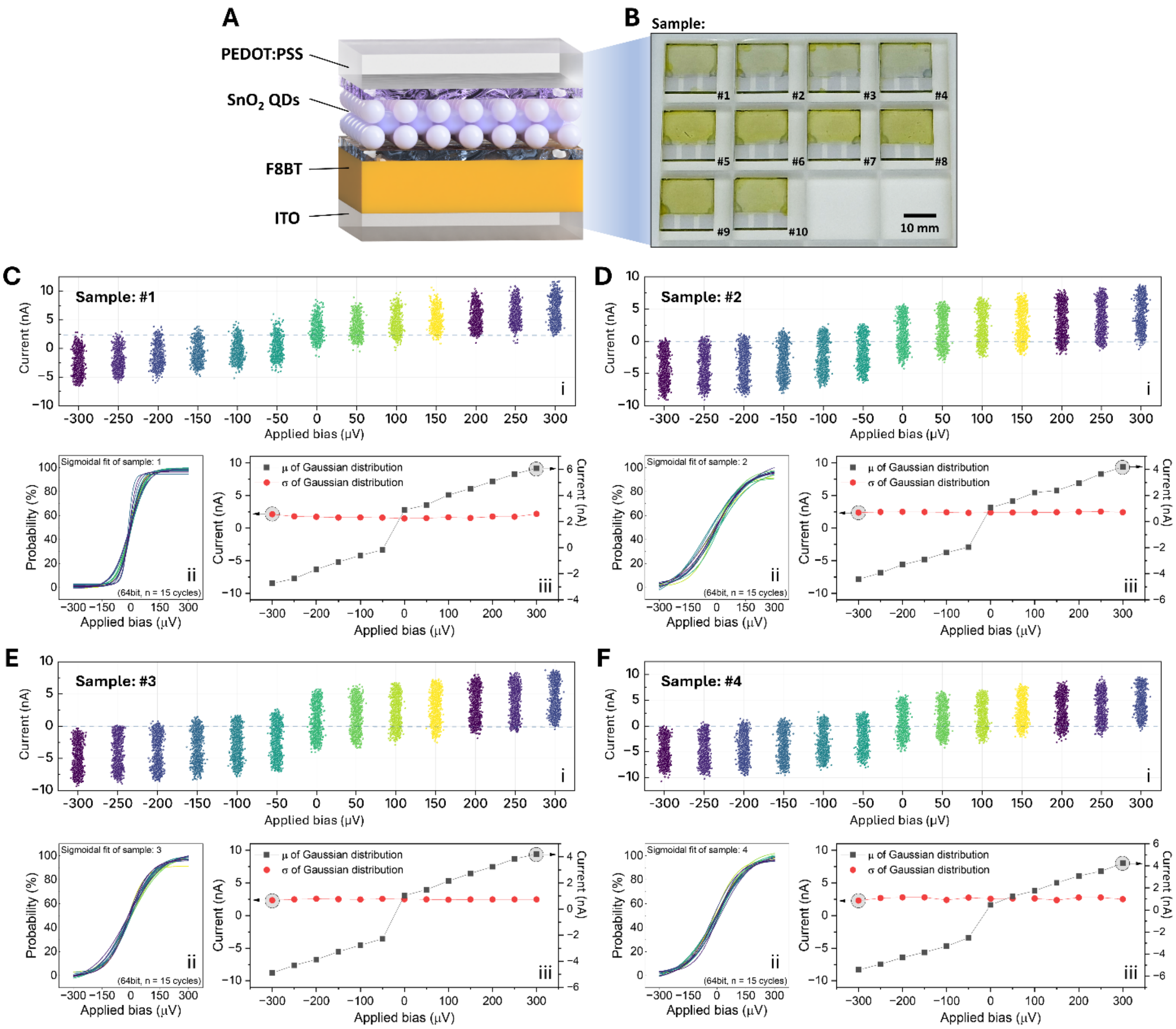
A
PEDOT:PSS
SnO_2 QDs
F8BT
ITO
B
Sample:
#1
#2
#3
#4
#5
#6
#7
#8
#9
#10
10 mm
C
Sample: #1
D
Sample: #2
E
Sample: #3
F
Sample: #4
Current (nA)
Applied bias (μV)
Probability (%)
Sigmoidal fit of sample: 1
Sigmoidal fit of sample: 2
Sigmoidal fit of sample: 3
Sigmoidal fit of sample: 4
(64bit, n = 15 cycles)
μ of Gaussian distribution
σ of Gaussian distribution
i
ii
iii

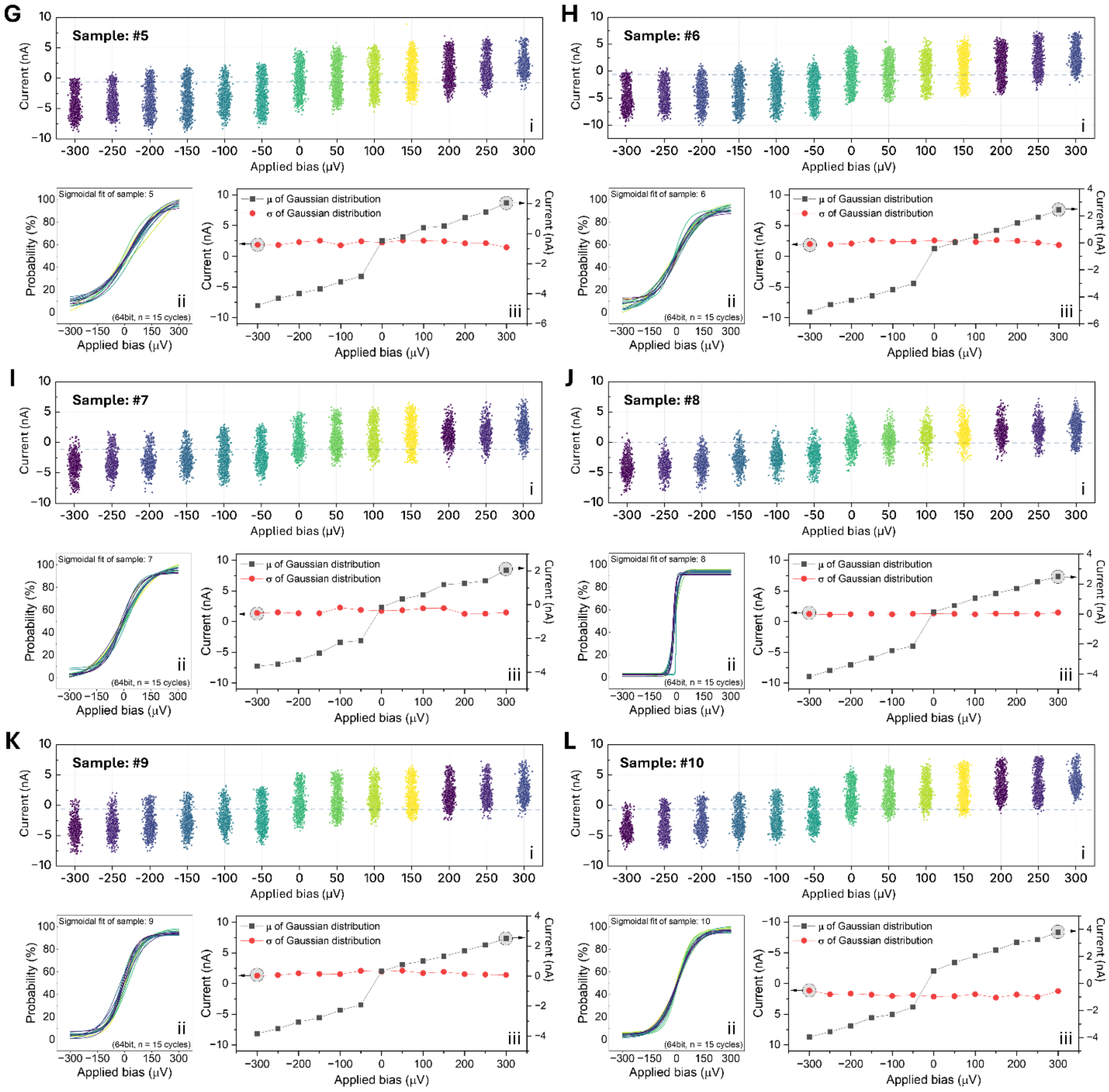

Fig. S5.

Reproducibility and device-to-device variation of LBP-bit devices. (A) Schematic illustration of the LBP-bit device structure. (B) Optical images of ten fabricated devices. (C-L) Results obtained from ten devices show that (i) Bias-current characteristics of each device, showing that the current distribution can be stably modulated by the applied bias. (ii) Results of repeated measurements, showing consistent sigmoid probability-voltage curves across devices. (iii) Gaussian distribution of the output current for each device, showing a gradual shift in the μ with the applied bias, while the σ remains nearly constant across all bias levels.

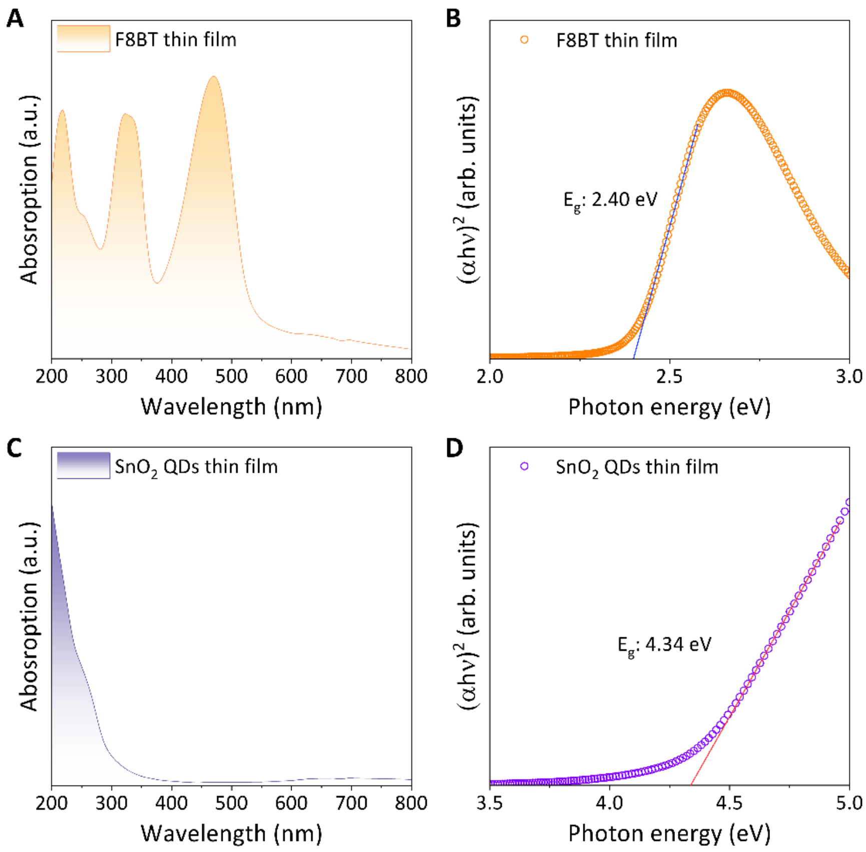

Fig. S6.

Optical absorption and Tauc plot analysis of F8BT and SnO_2 QDs. (A) Optical absorption spectrum of the F8BT film. (B) Tauc plot of F8BT derived from its optical absorption spectroscopy. (C) Optical absorption spectrum of the SnO_2 QD film. (D) Tauc plot of SnO_2 QDs derived from their optical absorption spectroscopy.

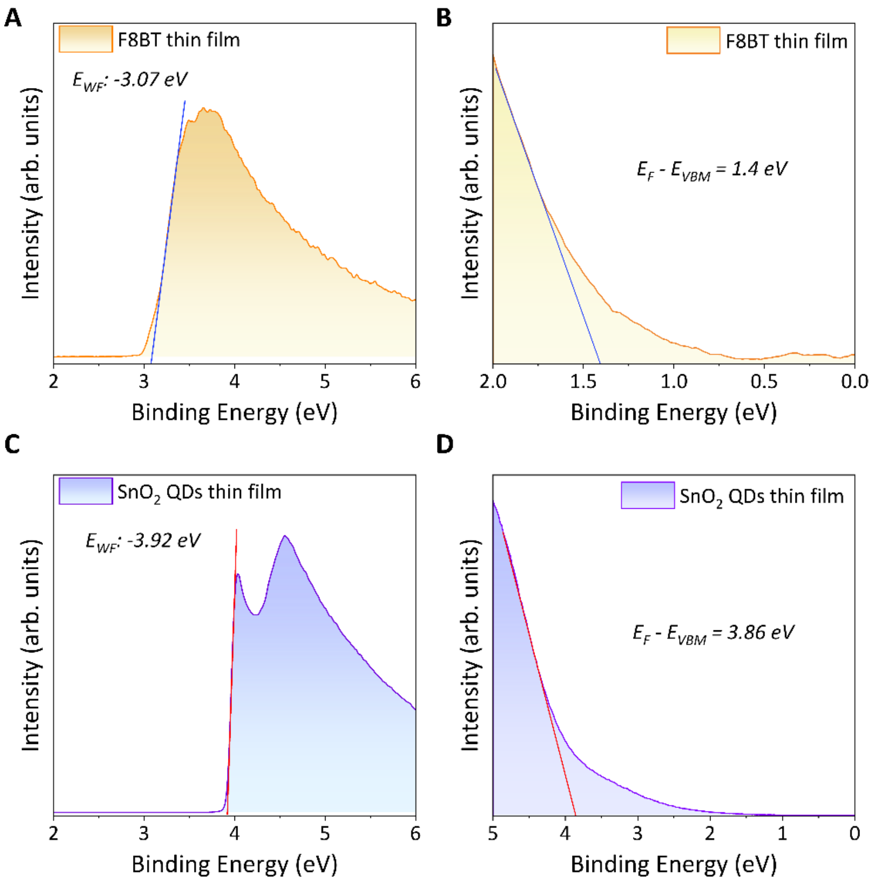

Fig. S7.

UPS analysis of F8BT and SnO_2 QDs. (A) Work function of the F8BT film determined from UPS cutoff energy. (B) VBM of the F8BT film obtained from the onset region. (C) UPS cutoff spectrum showing the work function of the SnO_2 QD film. (D) VBM of the SnO_2 QDs derived from the UPS onset region.

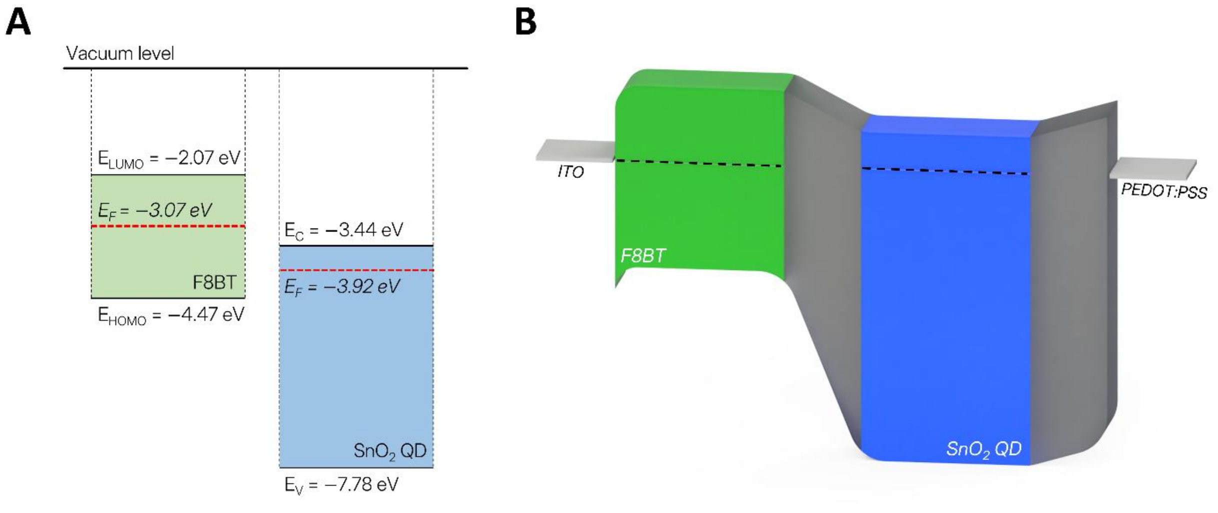

Fig. S8.

Energy band alignment and band bending at the heterojunction. (A) Flat-band energy diagrams of F8BT and SnO_2 QDs. (B) Schematic illustration of energy band bending resulting from Fermi level equilibration at the heterointerface.

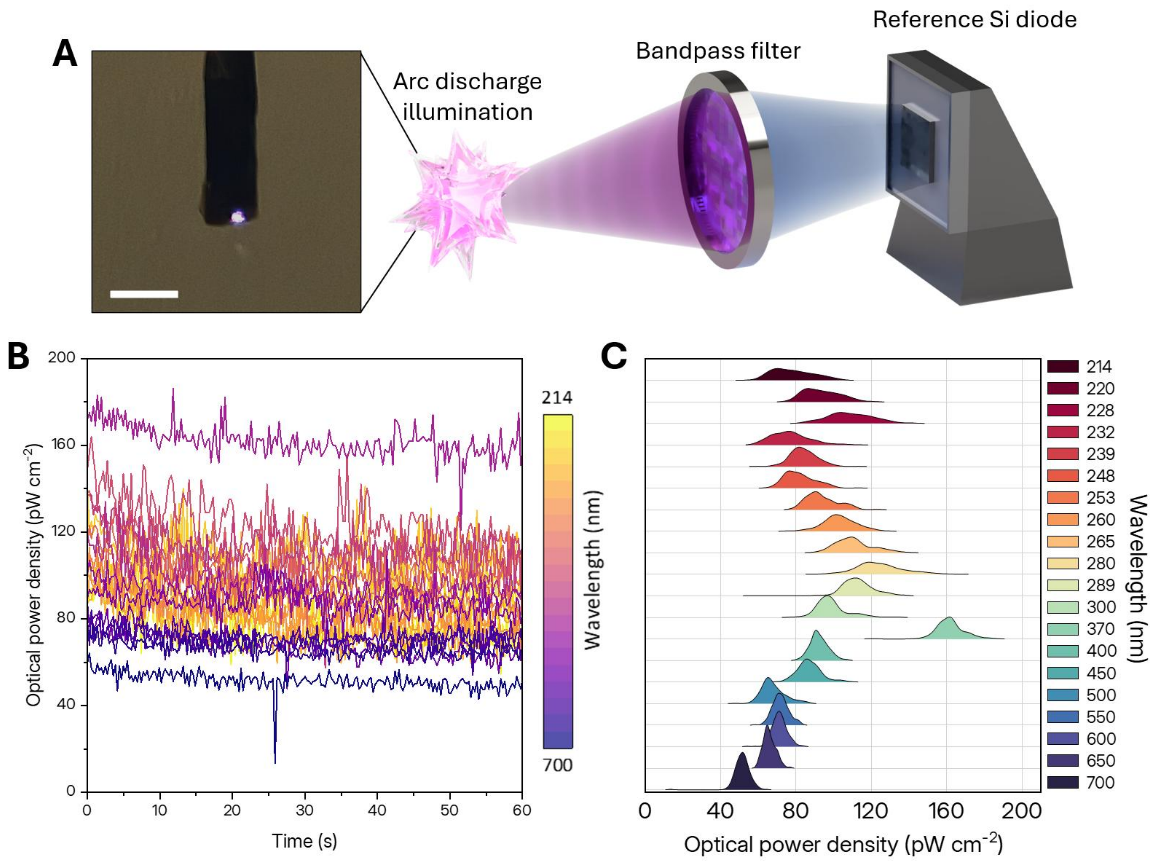

Fig. S9.

Wavelength-resolved characterization of the arc-discharge light source. (A) Photograph and schematic of the arc discharge light source and detection setup (scale bar: 3 cm). (B) Time-domain optical power traces measured through bandpass filters (214-700 nm). (C) Corresponding wavelength-dependent optical power density distributions.

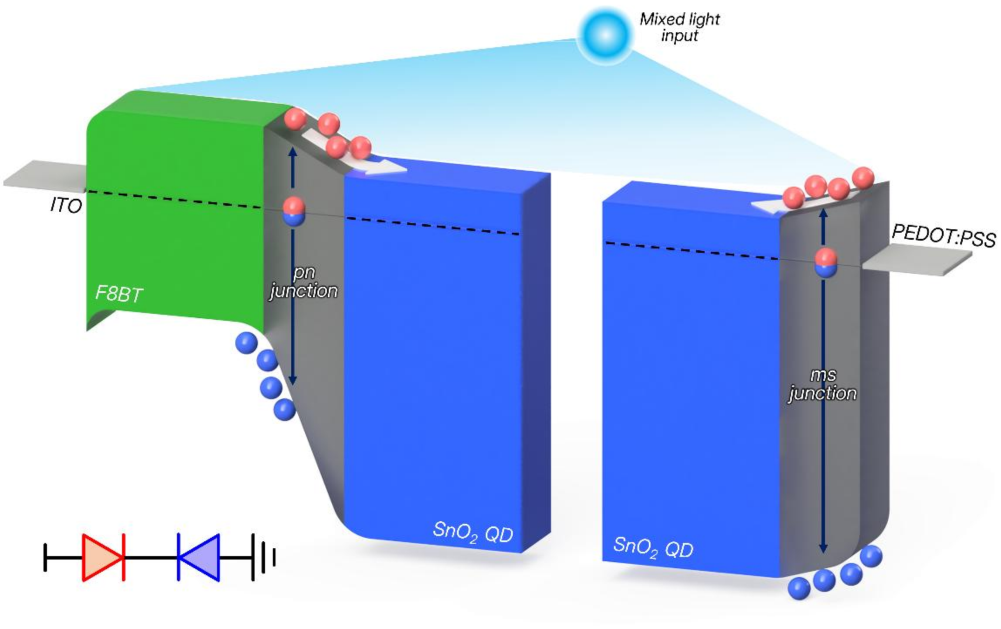

Fig. S10.

Schematic illustration of the unbiased states of the proposed device. Charge carrier dynamics under DIPS illumination in the proposed multi-heterojunction structure without external bias.

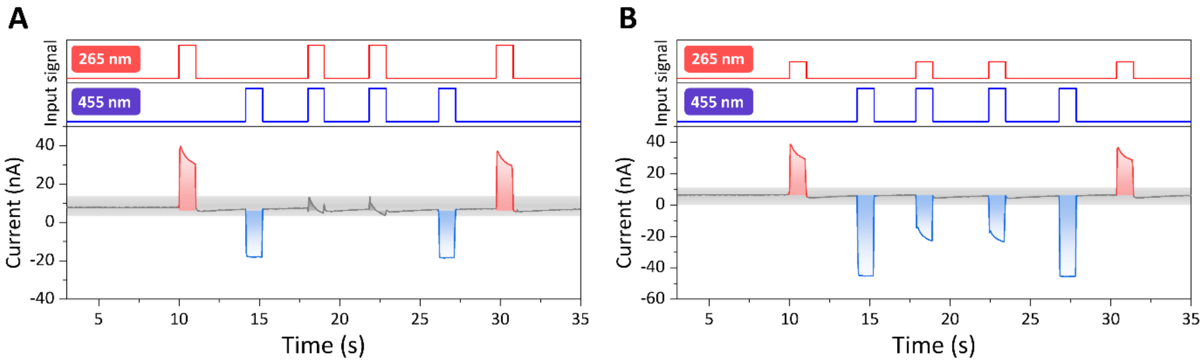

Fig. S11.

Selective optical activation of pn and ms junctions using dual-wavelength LEDs. (A) Photocurrent behavior under balanced illumination with 265 nm (121.38 μW cm^{-2}) and 455 nm (30.35 μW cm^{-2}) LEDs, yielding matched photocurrent levels at the ms and pn junctions. (B) Photocurrent behavior under imbalanced illumination with 265 nm (121.38 μW cm^{-2}) and 455 nm (75.15 μW cm^{-2}), generating unequal photocurrents at the two junctions due to differing light intensities.

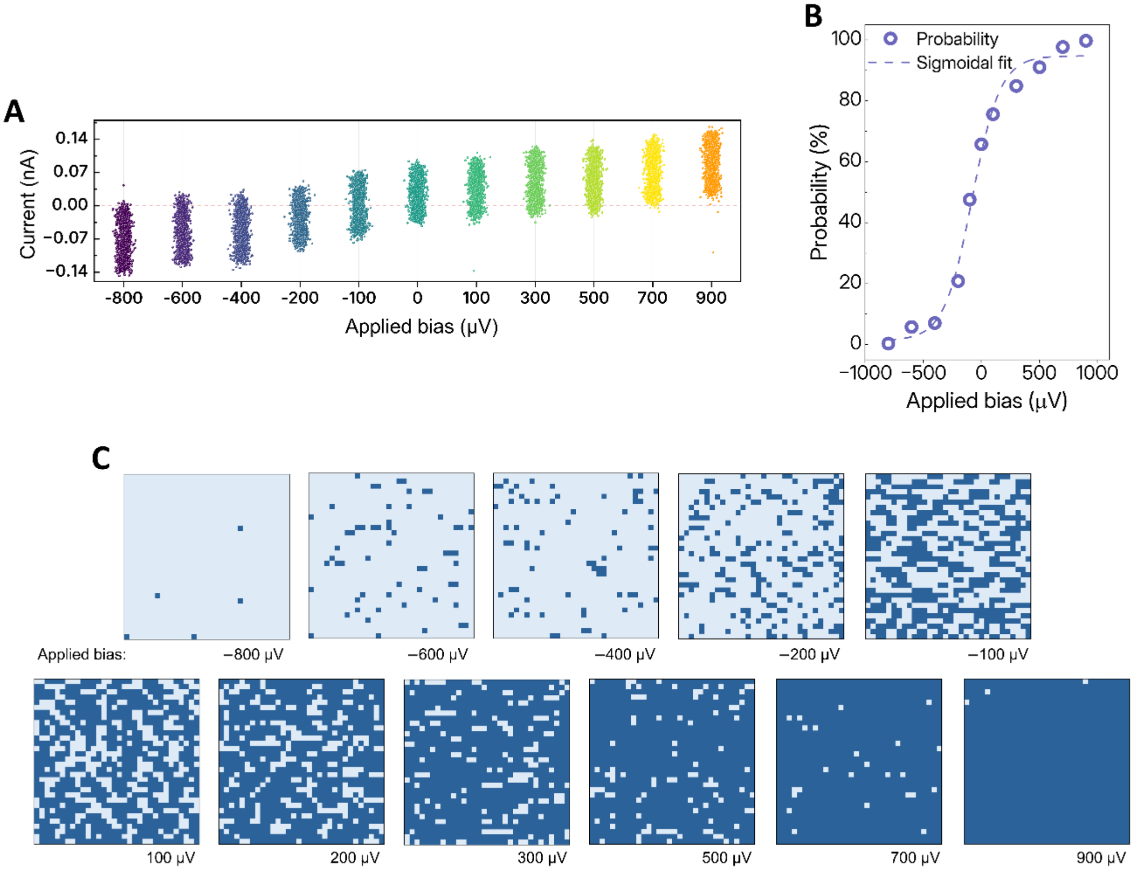

Fig. S12.

p-bit response under low DIPS intensity conditions. (A) Photocurrent modulation of the device under various bias conditions. (B) Sigmoidal curve showing the probability output of p-bits controlled by the applied bias voltage. (C) Mapping images of p-bit outputs for each voltage bias step.

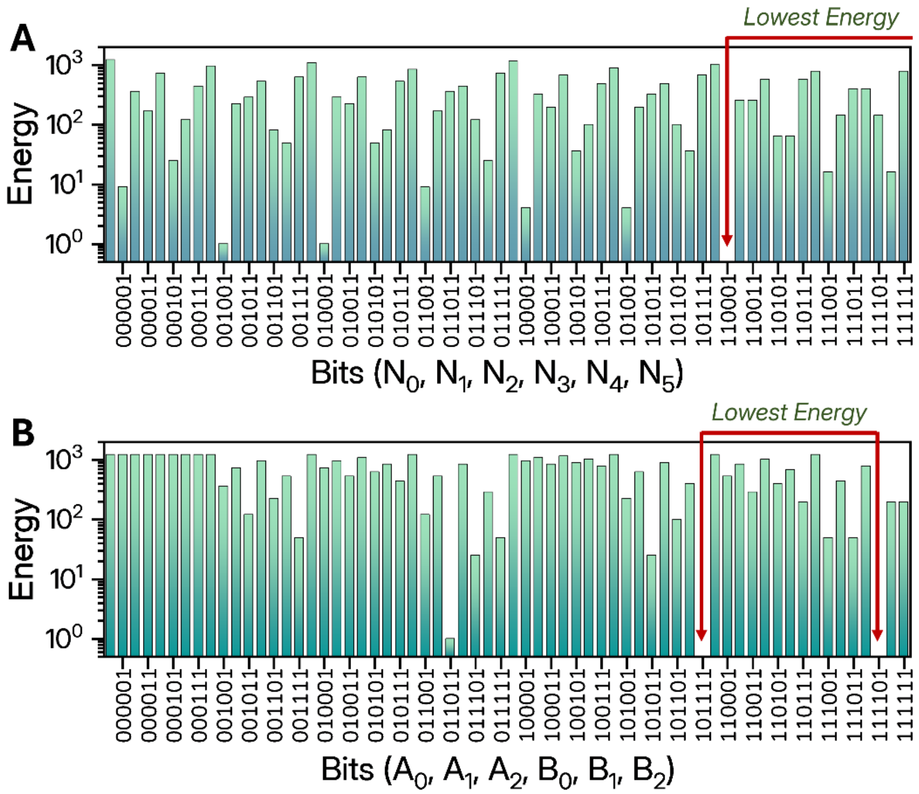

Fig. S13.

Energy of each configuration of the unfixed p-bits. (A) Energy of all unfixed p-bit configurations in the forward multiplication (A=7, B=5); minimum energy observed for N=35. (B) Energy of all unfixed p-bit configurations in the backward factorization (N=35); minimum energy observed for (A, B) = (5, 7) and (7, 5).

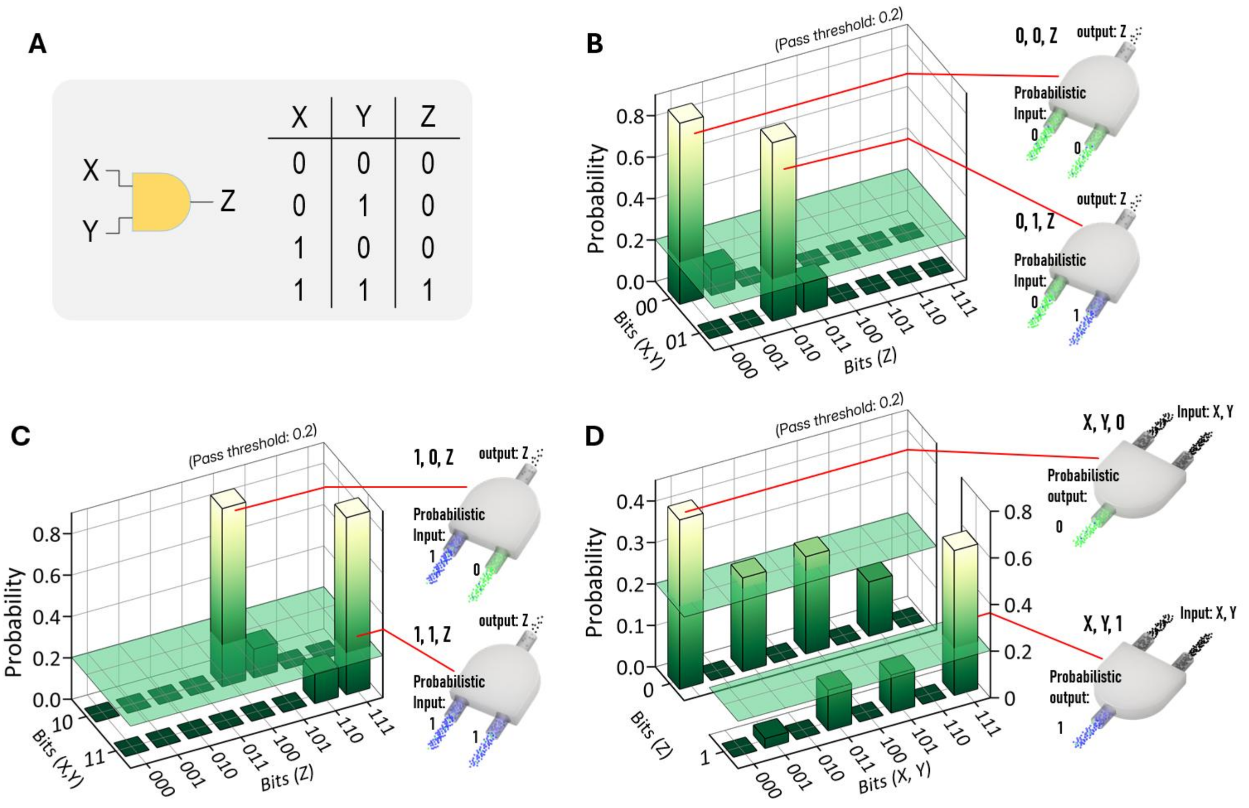

Fig. S14.

Inversible AND gate using three p-bits. (A) Truth table of AND gate. (B), (C) Forward operation (compute Z from fixed X, Y) showing the correct truth-table outcomes with probabilities over 0.8. (D) Backward operation (recover X, Y from fixed Z) in which only the valid input pairs for Z=0 and Z=1 exceed the pass threshold of 0.2.

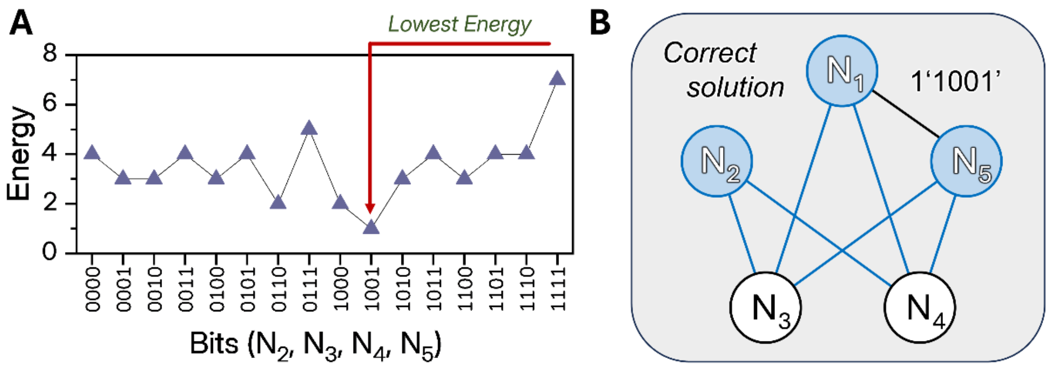

Fig. S15.

Max-Cut (5 nodes, 7 edges): energy landscape and solutions identified by p-computing. (A) Energy evaluated over all configurations of the four unfixed p-bits with $N_1=1$ fixed. The landscape exhibits a single global minimum. (B) Solution of the provided Max-Cut problem.

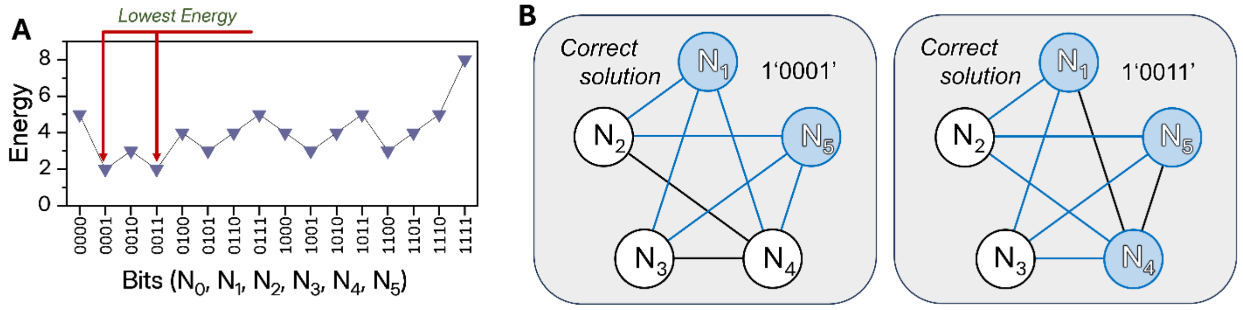

Fig. S16.

Max-Cut (5 nodes, 8 edges): energy landscape and solutions identified by p-computing. (A) Energy evaluated over all configurations of the four unfixed p-bits with N_1=1 fixed. The landscape exhibits two global minimums. (B) Solutions of the provided Max-Cut problem.

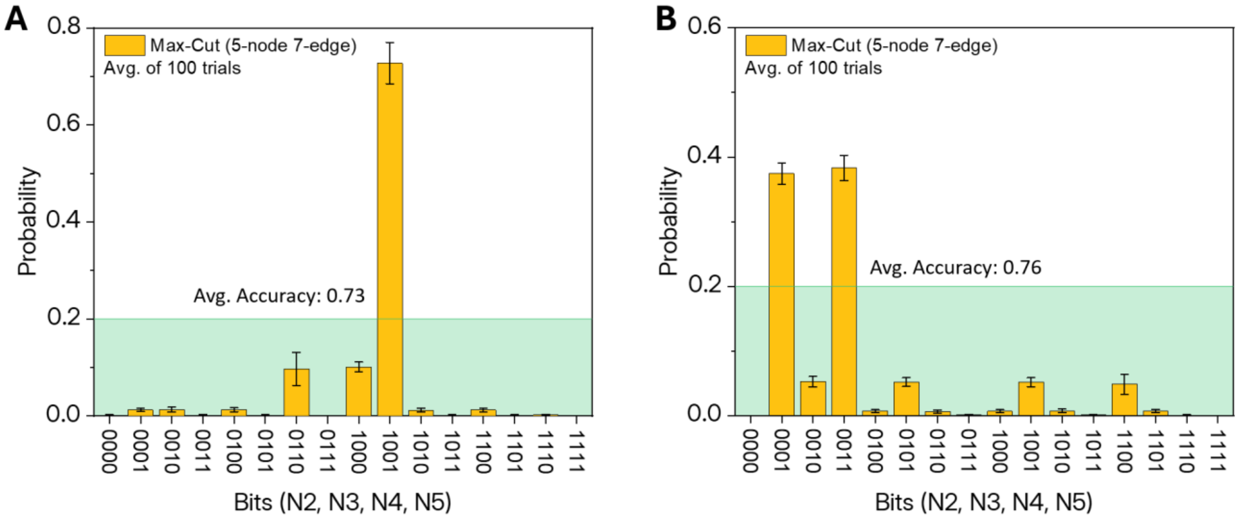

Fig. S17.

Results of Max-Cut trials, 100 cycles. (A) Outcome of 100 independent runs on the 5 nodes, 7 edges Max-Cut instance, together with the resulting standard deviation. (B) Repeated solving of the Max-Cut (5 nodes, 8 edges) problem 100 times and standard deviation of the results.

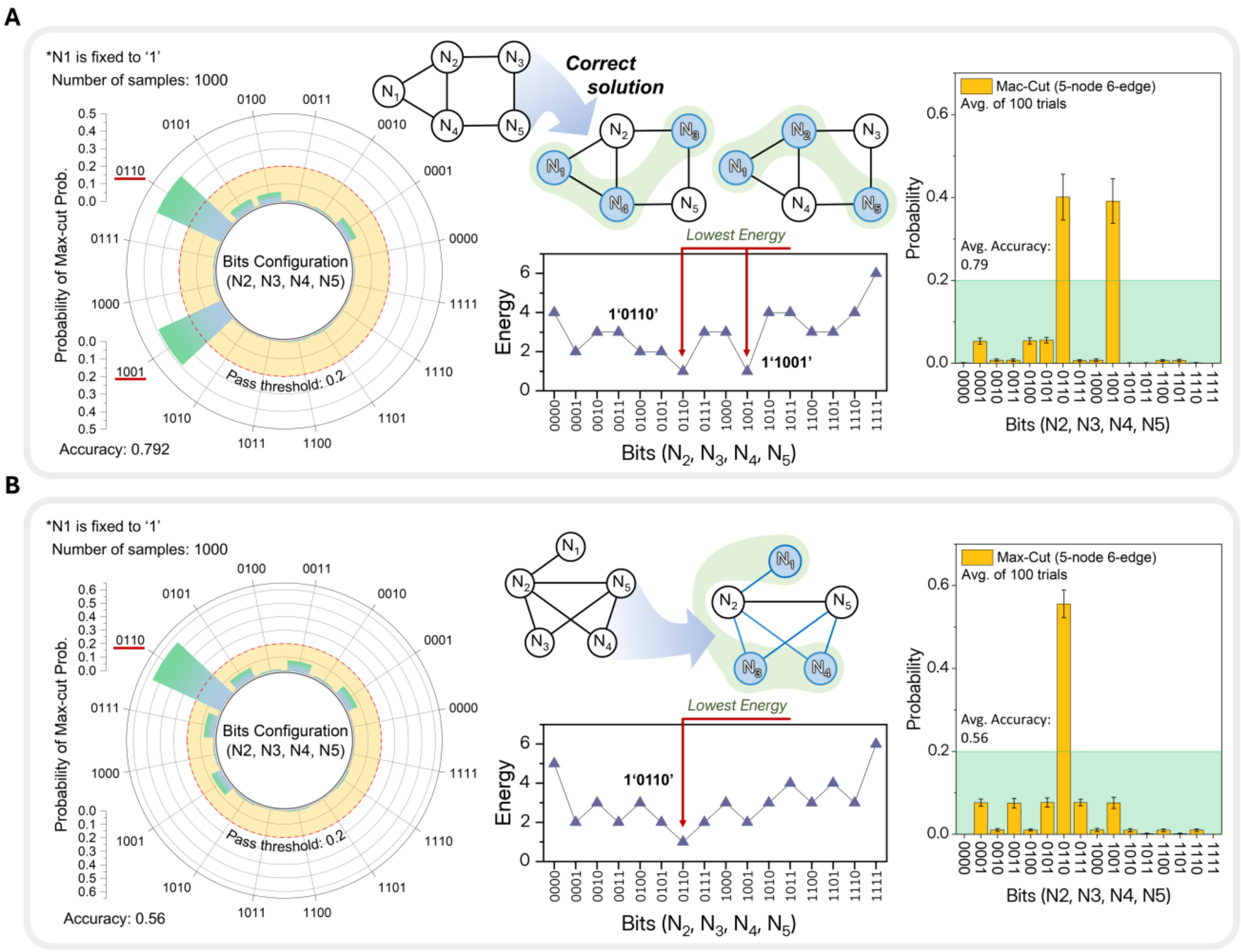

Fig. S18.

Additional Max-Cut instances solved by p-computing. Two additional Max-cut problems (5 nodes, 6 edges) solved using p-computing, with (A) two and (B) one global minima, respectively.

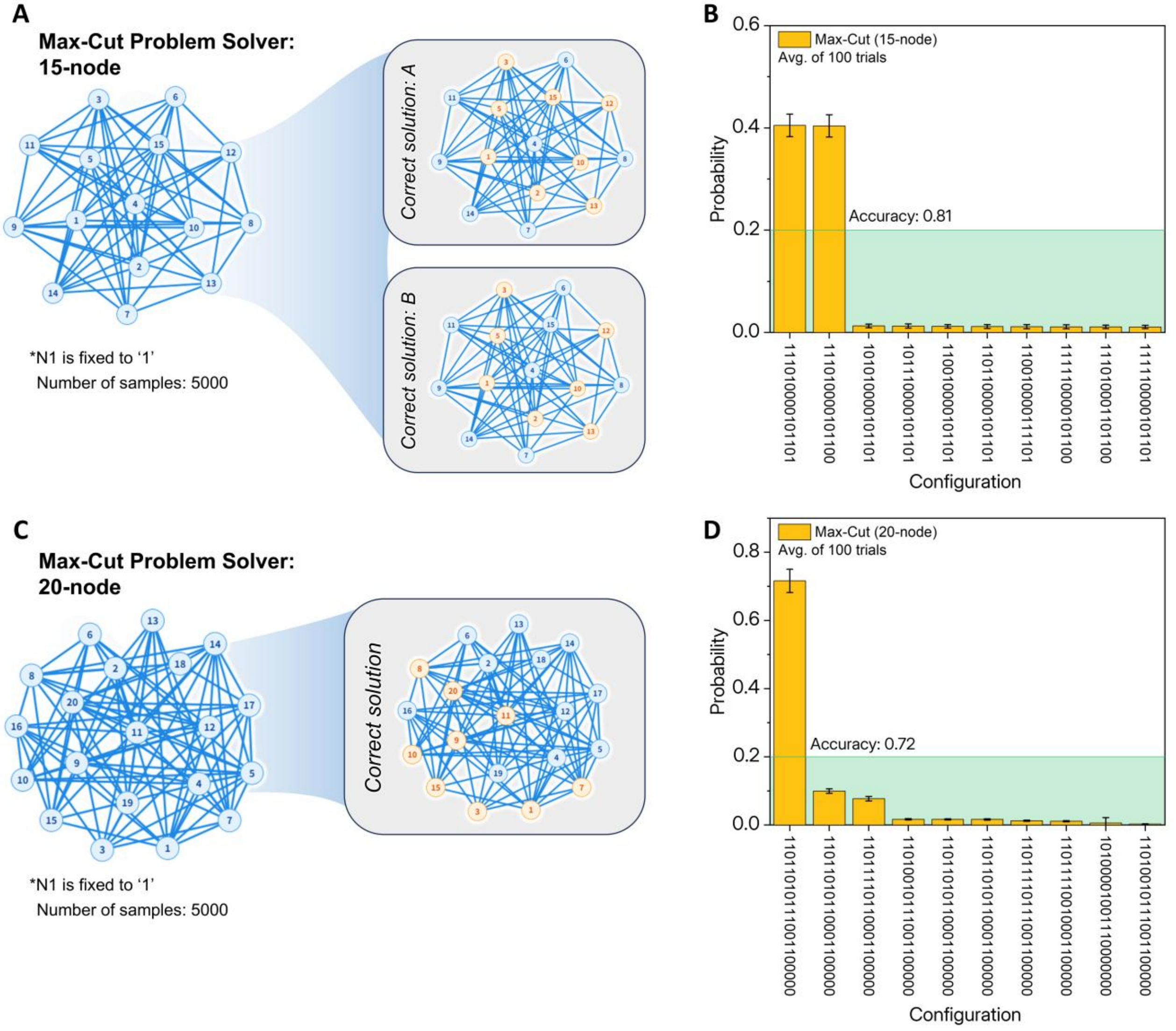

Fig. S19.

Extended probabilistic computing demonstration using a larger Max-Cut problem (15 and 20 nodes). (A) Graph structure of the 15-node Max-Cut problem and the configuration corresponding to the optimal solution (edge density 56%, corresponding to half of all possible 14C2 connections). (B) Solution probability distribution obtained from p-computing simulations for the 15-node problem. The optimal solution exceeds the pass-value threshold (0.2) with an average accuracy of 0.81. (C) Graph structure of the 20-node Max-Cut problem and configurations corresponding to the optimal solutions (edge density 48%, corresponding to half of all possible 19C2 connections). (D) Solution probability distribution obtained from p-computing simulations for the 20-node problem. The optimal solution exceeds the pass-value threshold (0.2) with an average accuracy of 0.72. Error bars represent the standard deviation.

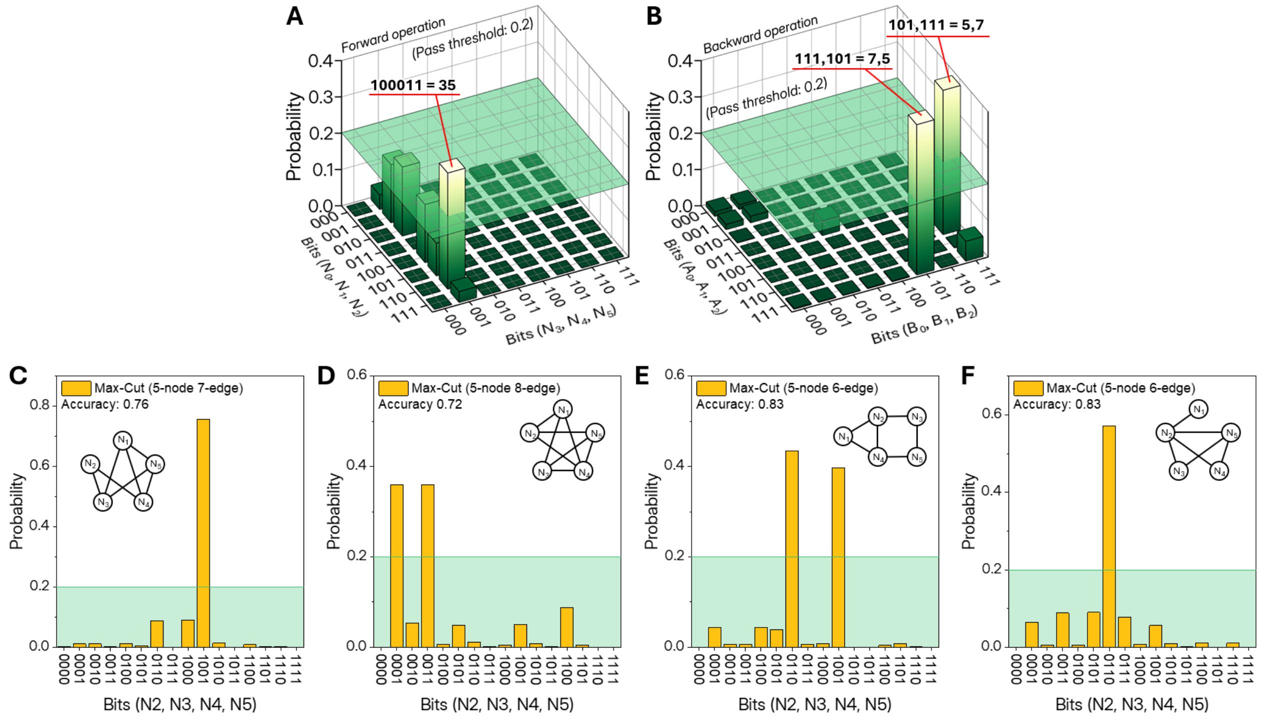

Fig. S20.

Multiplier and Max-Cut results with Supplementary Fig. 12. (A) Results of the forward (multiplication) and inverse (integer-factorization) operations of 3-bit by 3-bit binary multiplier. (B) Results of solving four Max-Cut instances (pass threshold = 0.2).

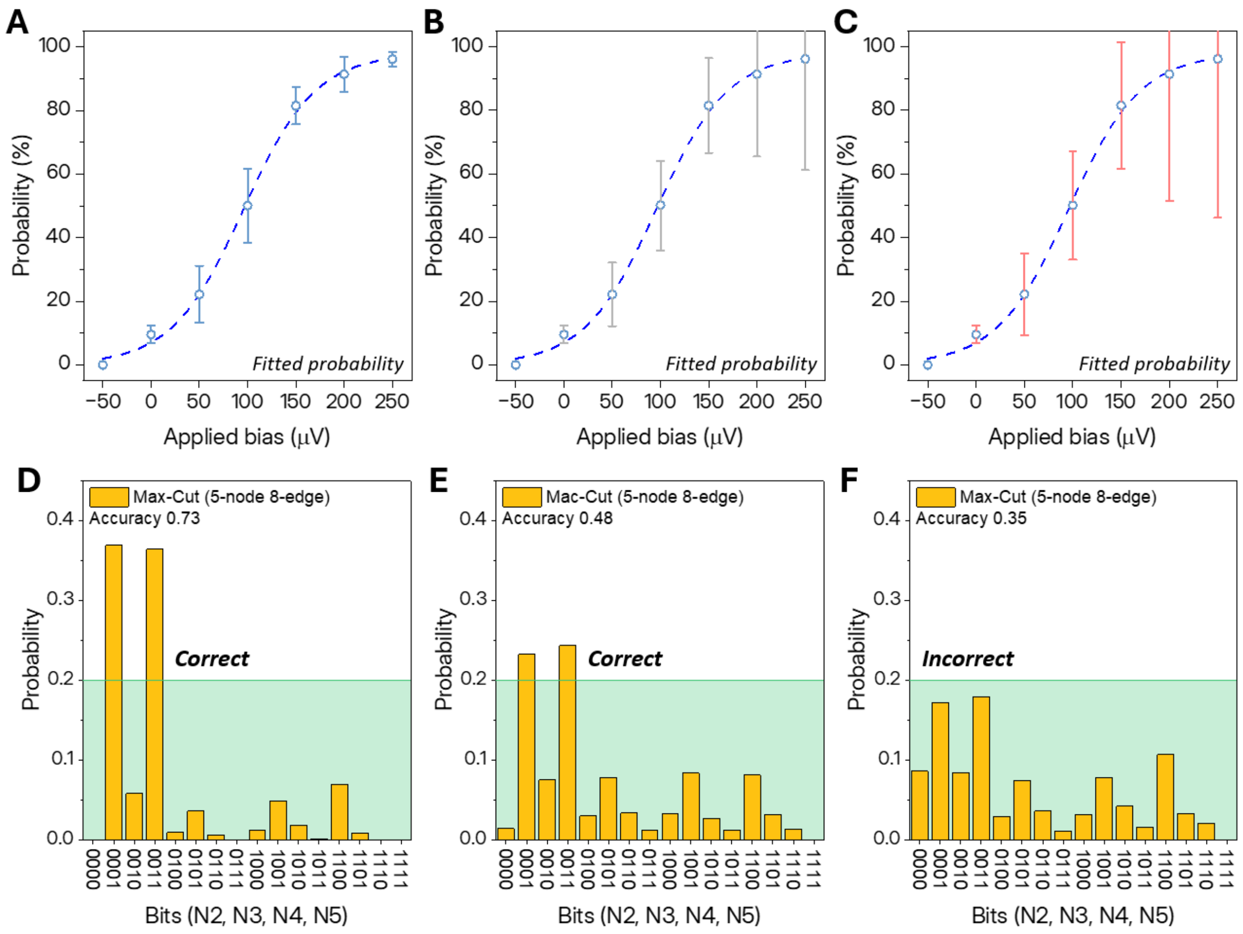

Fig. S21.

Effect of decoupled and coupled σ–bias behavior on p-computing accuracy. (A) Output probability characteristic of the LBP-bit device, exhibiting a shape-preserving mean shift with bias-invariant σ. (B, C) Hypothetical characteristics in which σ increases with applied bias, representing moderate and strong σ-bias coupling, respectively. (D) p-computing result for the 5-node Max-Cut instance of Fig. 5C obtained using the characteristic in (A). (E, F) Corresponding Max-Cut outcomes for the two hypothetical cases in (B) and (C).

Table S1.

Energy function and input function for the 3-bit by 3-bit binary multiplier.

Energy function (H)	$H = \{(32N_5 + 16N_4 + 8N_3 + 4N_2 + 2N_1 + N_0) - (4A_2 + 2A_1 + A_0)(4B_2 + 2B_1 + B_0)\}^2$	
Input function ($I_{X_i} = -\frac{\partial H}{\partial X_i}$)	A_2	$I_{A2} = -32\,A_0B_0B_1 - 64\,A_0B_0B_2 - 8\,A_0B_0 - 128\,A_0B_1B_2 - 32\,A_0B_1 - 128\,A_0B_2 - 64\,A_1B_0B_1 - 128\,A_1B_0B_2 - 16\,A_1B_0 - 256\,A_1B_1B_2 - 64\,A_1B_1 - 256\,A_1B_2 - 64\,B_0B_1 - 128\,B_0B_2 + 8\,B_0N_0 + 16\,B_0N_1 + 32\,B_0N_2 + 64\,B_0N_3 + 128\,B_0N_4 + 256\,B_0N_5 - 16\,B_0 - 256\,B_1B_2 + 16\,B_1N_0 + 32\,B_1N_1 + 64\,B_1N_2 + 128\,B_1N_3 + 256\,B_1N_4 + 512\,B_1N_5 - 64\,B_1 + 32\,B_2N_0 + 64\,B_2N_1 + 128\,B_2N_2 + 256\,B_2N_3 + 512\,B_2N_4 + 1024\,B_2N_5 - 256\,B_2$
	A_1	$I_{A1} = -16\,A_0B_0B_1 - 32\,A_0B_0B_2 - 4\,A_0B_0 - 64\,A_0B_1B_2 - 16\,A_0B_1 - 64\,A_0B_2 - 64\,A_2B_0B_1 - 128\,A_2B_0B_2 - 16\,A_2B_0 - 256\,A_2B_1B_2 - 64\,A_2B_1 - 256\,A_2B_2 - 16\,B_0B_1 - 32\,B_0B_2 + 4\,B_0N_0 + 8\,B_0N_1 + 16\,B_0N_2 + 32\,B_0N_3 + 64\,B_0N_4 + 128\,B_0N_5 - 4\,B_0 - 64\,B_1B_2 + 8\,B_1N_0 + 16\,B_1N_1 + 32\,B_1N_2 + 64\,B_1N_3 + 128\,B_1N_4 + 256\,B_1N_5 - 16\,B_1 + 16\,B_2N_0 + 32\,B_2N_1 + 64\,B_2N_2 + 128\,B_2N_3 + 256\,B_2N_4 + 512\,B_2N_5 - 64\,B_2$
	A_0	$I_{A0} = -16\,A_1B_0B_1 - 32\,A_1B_0B_2 - 4\,A_1B_0 - 64\,A_1B_1B_2 - 16\,A_1B_1 - 64\,A_1B_2 - 32\,A_2B_0B_1 - 64\,A_2B_0B_2 - 8\,A_2B_0 - 128\,A_2B_1B_2 - 32\,A_2B_1 - 128\,A_2B_2 - 4\,B_0B_1 - 8\,B_0B_2 + 2\,B_0N_0 + 4\,B_0N_1 + 8\,B_0N_2 + 16\,B_0N_3 + 32\,B_0N_4 + 64\,B_0N_5 - B_0 - 16\,B_1B_2 + 4\,B_1N_0 + 8\,B_1N_1 + 16\,B_1N_2 + 32\,B_1N_3 + 64\,B_1N_4 + 128\,B_1N_5 - 4\,B_1 + 8\,B_2N_0 + 16\,B_2N_1 + 32\,B_2N_2 + 64\,B_2N_3 + 128\,B_2N_4 + 256\,B_2N_5 - 16\,B_2$
	B_2	$I_{B2} = -32\,A_0A_1B_0 - 64\,A_0A_1B_1 - 64\,A_0A_1 - 64\,A_0A_2B_0 - 128\,A_0A_2B_1 - 128\,A_0A_2 - 8\,A_0B_0 - 16\,A_0B_1 + 8\,A_0N_0 + 16\,A_0N_1 + 32\,A_0N_2 + 64\,A_0N_3 + 128\,A_0N_4 + 256\,A_0N_5 - 16\,A_0 - 128\,A_1A_2B_0 - 256\,A_1A_2B_1 - 256\,A_1A_2 - 32\,A_1B_0 - 64\,A_1B_1 + 16\,A_1N_0 + 32\,A_1N_1 + 64\,A_1N_2 + 128\,A_1N_3 + 256\,A_1N_4 + 512\,A_1N_5 - 64\,A_1 - 128\,A_2B_0 - 256\,A_2B_1 + 32\,A_2N_0 + 64\,A_2N_1 + 128\,A_2N_2 + 256\,A_2N_3 + 512\,A_2N_4 + 1024\,A_2N_5 - 256\,A_2$
	B_1	$I_{B1} = -16\,A_0A_1B_0 - 64\,A_0A_1B_2 - 16\,A_0A_1 - 32\,A_0A_2B_0 - 128\,A_0A_2B_2 - 32\,A_0A_2 - 4\,A_0B_0 - 16\,A_0B_2 + 4\,A_0N_0 + 8\,A_0N_1 + 16\,A_0N_2 + 32\,A_0N_3 + 64\,A_0N_4 + 128\,A_0N_5 - 4\,A_0 - 64\,A_1A_2B_0 - 256\,A_1A_2B_2 - 64\,A_1A_2 - 16\,A_1B_0 - 64\,A_1B_2 + 8\,A_1N_0 + 16\,A_1N_1 + 32\,A_1N_2 + 64\,A_1N_3 + 128\,A_1N_4 + 256\,A_1N_5 - 16\,A_1 - 64\,A_2B_0 - 256\,A_2B_2 + 16\,A_2N_0 + 32\,A_2N_1 + 64\,A_2N_2 + 128\,A_2N_3 + 256\,A_2N_4 + 512\,A_2N_5 - 64\,A_2$
	B_0	$I_{B0} = -16\,A_0A_1B_1 - 32\,A_0A_1B_2 - 4\,A_0A_1 - 32\,A_0A_2B_1 - 64\,A_0A_2B_2 - 8\,A_0A_2 - 4\,A_0B_1 - 8\,A_0B_2 + 2\,A_0N_0 + 4\,A_0N_1 + 8\,A_0N_2 + 16\,A_0N_3 + 32\,A_0N_4 + 64\,A_0N_5 - A_0 - 64\,A_1A_2B_1 - 128\,A_1A_2B_2 - 16\,A_1A_2 - 16\,A_1B_1 - 32\,A_1B_2 + 4\,A_1N_0 + 8\,A_1N_1 + 16\,A_1N_2 + 32\,A_1N_3 + 64\,A_1N_4 + 128\,A_1N_5 - 4\,A_1 - 64\,A_2B_1 - 128\,A_2B_2 + 8\,A_2N_0 + 16\,A_2N_1 + 32\,A_2N_2 + 64\,A_2N_3 + 128\,A_2N_4 + 256\,A_2N_5 - 16\,A_2$
	N_5	$I_{N5} = 64\,A_0B_0 + 128\,A_0B_1 + 256\,A_0B_2 + 128\,A_1B_0 + 256\,A_1B_1 + 512\,A_1B_2 + 256\,A_2B_0 + 512\,A_2B_1 + 1024\,A_2B_2 - 64\,N_0 - 128\,N_1 - 256\,N_2 - 512\,N_3 - 1024\,N_4 - 1024$
	N_4	$I_{N4} = 32\,A_0B_0 + 64\,A_0B_1 + 128\,A_0B_2 + 64\,A_1B_0 + 128\,A_1B_1 + 256\,A_1B_2 + 128\,A_2B_0 + 256\,A_2B_1 + 512\,A_2B_2 - 32\,N_0 - 64\,N_1 - 128\,N_2 - 256\,N_3 - 1024\,N_5 - 256$
	N_3	$I_{N3} = 16\,A_0B_0 + 32\,A_0B_1 + 64\,A_0B_2 + 32\,A_1B_0 + 64\,A_1B_1 + 128\,A_1B_2 + 64\,A_2B_0 + 128\,A_2B_1 + 256\,A_2B_2 - 16\,N_0 - 32\,N_1 - 64\,N_2 - 256\,N_4 - 512\,N_5 - 64$
	N_2	$I_{N2} = 8\,A_0B_0 + 16\,A_0B_1 + 32\,A_0B_2 + 16\,A_1B_0 + 32\,A_1B_1 + 64\,A_1B_2 + 32\,A_2B_0 + 64\,A_2B_1 + 128\,A_2B_2 - 8\,N_0 - 16\,N_1 - 64\,N_3 - 128\,N_4 - 256\,N_5 - 16$
	N_1	$I_{N1} = 4\,A_0B_0 + 8\,A_0B_1 + 16\,A_0B_2 + 8\,A_1B_0 + 16\,A_1B_1 + 32\,A_1B_2 + 16\,A_2B_0 + 32\,A_2B_1 + 64\,A_2B_2 - 4\,N_0 - 16\,N_2 - 32\,N_3 - 64\,N_4 - 128\,N_5 - 4$
	N_0	$I_{N0} = 2\,A_0B_0 + 4\,A_0B_1 + 8\,A_0B_2 + 4\,A_1B_0 + 8\,A_1B_1 + 16\,A_1B_2 + 8\,A_2B_0 + 16\,A_2B_1 + 32\,A_2B_2 - 4\,N_1 - 8\,N_2 - 16\,N_3 - 32\,N_4 - 64\,N_5 - 1$

Table S2.

Energy function and input function for the Max-Cut problem (Fig. 5A)

Energy function (H)	$H = \sum_{(i,j)\in E} \left(2N_iN_j - N_i - N_j + 1\right)$ ($E = \{(1,3),\ (1,4),\ (1,5),\ (2,3),\ (2,4),\ (3,5),\ (4,5)\}$)	
Input function ($I_{X_i} = -\frac{\partial H}{\partial X_i}$)	N_1	$I_{N_1} = -2N_3 - 2N_4 - 2N_5 + 3$
	N_2	$I_{N_2} = -2N_3 - 2N_4 + 2$
	N_3	$I_{N_3} = -2N_1 - 2N_2 - 2N_5 + 3$
	N_4	$I_{N_4} = -2N_1 - 2N_2 - 2N_5 + 3$
	N_5	$I_{N_5} = -2N_1 - 2N_3 - 2N_4 + 3$

Table S3.

Energy function and input function for the Max-Cut problem (Fig. 5C)

Energy function (H)	$H = \sum_{(i,j)\in E} \left(2N_iN_j - N_i - N_j + 1\right)$ ($E = \{(1,2),\ (1,3),\ (1,4),\ (2,4),\ (2,5),\ (3,4),\ (3,5),\ (4,5)\}$)	
Input function ($I_{X_i} = -\frac{\partial H}{\partial X_i}$)	N_1	$I_{N_1} = -2N_2 - 2N_3 - 2N_4 + 3$
	N_2	$I_{N_2} = -2N_1 - 2N_4 - 2N_5 + 3$
	N_3	$I_{N_3} = -2N_1 - 2N_4 - 2N_5 + 3$
	N_4	$I_{N_4} = -2N_1 - 2N_2 - 2N_3 - 2N_5 + 4$
	N_5	$I_{N_5} = -2N_2 - 2N_3 - 2N_4 + 3$

Table S4.

Energy function and input function for the Max-Cut problem (Fig. S13A)

Energy function (H)	$H = \sum_{(i,j)\in E} \left(2N_iN_j - N_i - N_j + 1\right)$ ($E = \{(1,2),\ (1,4),\ (2,3),\ (2,4),\ (3,5),\ (4,5)\}$)	
Input function ($I_{X_i} = -\frac{\partial H}{\partial X_i}$)	N_1	$I_{N_1} = -2N_2 - 2N_4 + 2$
	N_2	$I_{N_2} = -2N_1 - 2N_3 - 2N_4 + 3$
	N_3	$I_{N_3} = -2N_2 - 2N_5 + 2$
	N_4	$I_{N_4} = -2N_1 - 2N_2 - 2N_5 + 3$
	N_5	$I_{N_5} = -2N_3 - 2N_4 + 2$

Table S5.

Energy function and input function for the Max-Cut problem (Fig. S13B)

Energy function (H)	$H = \sum_{(i,j)\in E} \left(2N_iN_j - N_i - N_j + 1\right)$ ($E = \{(1,2),\ (2,3),\ (2,4),\ (2,5),\ (3,5),\ (4,5)\}$)	
Input function ($I_{X_i} = -\frac{\partial H}{\partial X_i}$)	N_1	$I_{N_1} = -2N_2 + 1$
	N_2	$I_{N_2} = -2N_1 - 2N_3 - 2N_4 - 2N_5 + 4$
	N_3	$I_{N_3} = -2N_2 - 2N_5 + 2$
	N_4	$I_{N_4} = -2N_2 - 2N_5 + 2$
	N_5	$I_{N_5} = -2N_2 - 2N_3 - 2N_4 + 3$
